# Biological Mechanisms for Learning: A Computational Model of Olfactory Learning in the *Manduca sexta* Moth, With Applications to Neural Nets

**DOI:** 10.3389/fncom.2018.00102

**Published:** 2018-12-19

**Authors:** Charles B. Delahunt, Jeffrey A. Riffell, J. Nathan Kutz

**Affiliations:** ^1^Department of Electrical Engineering, University of Washington, Seattle, WA, United States; ^2^Computational Neuroscience Center, University of Washington, Seattle, WA, United States; ^3^Department of Biology, University of Washington, Seattle, WA, United States; ^4^Department of Applied Mathematics, University of Washington, Seattle, WA, United States

**Keywords:** antennal lobe, mushroom body, learning, olfaction, octopamine, sparsity, Hebbian, neural nets

## Abstract

The insect olfactory system, which includes the antennal lobe (AL), mushroom body (MB), and ancillary structures, is a relatively simple neural system capable of learning. Its structural features, which are widespread in biological neural systems, process olfactory stimuli through a cascade of networks where large dimension shifts occur from stage to stage and where sparsity and randomness play a critical role in coding. Learning is partly enabled by a neuromodulatory reward mechanism of octopamine stimulation of the AL, whose increased activity induces synaptic weight updates in the MB through Hebbian plasticity. Enforced sparsity in the MB focuses Hebbian growth on neurons that are the most important for the representation of the learned odor. Based upon current biophysical knowledge, we have constructed an end-to-end computational firing-rate model of the *Manduca sexta* moth olfactory system which includes the interaction of the AL and MB under octopamine stimulation. Our model is able to robustly learn new odors, and neural firing rates in our simulations match the statistical features of *in vivo* firing rate data. From a biological perspective, the model provides a valuable tool for examining the role of neuromodulators, like octopamine, in learning, and gives insight into critical interactions between sparsity, Hebbian growth, and stimulation during learning. Our simulations also inform predictions about structural details of the olfactory system that are not currently well-characterized. From a machine learning perspective, the model yields bio-inspired mechanisms that are potentially useful in constructing neural nets for rapid learning from very few samples. These mechanisms include high-noise layers, sparse layers as noise filters, and a biologically-plausible optimization method to train the network based on octopamine stimulation, sparse layers, and Hebbian growth.

## 1. Introduction

Learning is a vital function of biological neural networks, yet the underlying mechanisms responsible for robust and rapid learning are not well understood. The insect olfactory network, and the moth's olfactory network (MON) in particular (e.g., in the *Manduca sexta* moth), is a comparatively simple biological neural network capable of learning (Daly et al., 2001; Riffell et al., 2008), and makes an ideal model organism for characterizing the mechanics of learning. It is amenable to interrogation through experimental neural recordings of key, well-understood structural components including the antennal lobe (AL) (Wilson, 2008) and mushroom body (MB) (Campbell and Turner, 2010). In addition, the AL-MB contain many structural motifs that are widespread in biological neural systems. These motifs include: (i) the use of neuromodulators (octopamine and dopamine) in learning (Dacks et al., 2012), (ii) a cascading networks structure (Masse et al., 2009), (iii) large changes in dimensionality (i.e., numbers of neurons) between networks (Laurent, 2002), (iv) sparse encodings of data in high-dimensional networks (Honegger et al., 2011), (v) random connections (Caron, 2013), and (vi) the presence of noisy signals (Galizia, 2014). Bio-inspired design principles suggest that each of the features has high value to the olfactory system. The mechanism of octopamine/dopamine release during learning is of particular interest, since it is not well-understood how this stimulation promotes the construction of new sparse codes in the MB.

In this work, we build a computational model of the moth olfactory network, including both AL and MB, that is closely aligned with both the known biophysics of the moth AL-MB and *in vivo* neural firing rate data, and that includes the dynamics of octopamine stimulation. We then run simulations to investigate how the system components interact to learn new odors. When building our Network Model we have consulted the literature, subject to a caveat: Moths and flies are similar enough that findings in flies (*Drosophila*) can generally be transferred to the moth; but locusts and honeybees are more complex, and some findings in these insects do not safely transfer, while other findings are general enough to readily apply (Riffell et al., 2009a).

There exist several computational models based on the insect brain (García-Sanchez and Huerta, 2003; Nowotny et al., 2005; Jortner et al., 2007; Huerta and Nowotny, 2009; Nowotny, 2009; Arena et al., 2013; Bazhenov et al., 2013; Mosqueiro and Huerta, 2014; Faghihi et al., 2017; Peng and Chittka, 2017; Roper et al., 2017). Because the MB is central to memory, these models focus on the sparsely-firing, high-dimensional MB plus readout neuron(s), leaving aside the AL or treating it as a “pass-through.” Some of these models incorporate forms of Hebbian plasticity (Nowotny et al., 2005; Huerta and Nowotny, 2009; Nowotny, 2009; Bazhenov et al., 2013; Peng and Chittka, 2017). In general these models are not closely tied to a particular organism (though they are usually inspired by locusts or honeybees), so they are “top-down” designs, allowing freedom with model parameters in the service of capturing general behaviors (indeed, Peng and Chittka, 2017 points out the advantages of this more general approach).

Key findings of these models include: The value (for class separation) of the fan-out into the high-dimensional, sparsely-firing MB; theoretical calculations of parameters such as optimal AL → MB connectivity (García-Sanchez and Huerta, 2003; Nowotny, 2009); the value of random neural connectivity (Nowotny, 2009; Peng and Chittka, 2017); the ability of the simple MB structure to capture complex behaviors (Bazhenov et al., 2013; Peng and Chittka, 2017; Roper et al., 2017); robust performance over wide tuning parameter ranges (Huerta and Nowotny, 2009; Roper et al., 2017); and the generalized learning skills of the insect MB, given a Hebbian update mechanism (Huerta and Nowotny, 2009; Arena et al., 2013; Mosqueiro and Huerta, 2014; Faghihi et al., 2017; Peng and Chittka, 2017).

Our computational model of learning in the MON is distinct in four key ways from these general, MB-focused studies. (i) We model the architecture and neural dynamics of the whole system. This includes detailed internal wiring of the AL, the MB, inhibition from the Lateral Horn, octopamine stimulation during learning, Hebbian plasticity, and an extrinsic (readout) neuron downstream. Linking careful models of the AL and MB fills a particular gap in the literature called out by Mosqueiro and Huerta (2014). (ii) We include octopamine stimulation in the dynamics equations. The neuromodulator octopamine (similarly dopamine) is essential to learning in the insect olfactory network (Hammer and Menzel, 1995, 1998), but it has not (to our knowledge) been incorporated into a computational model. Thanks to a unique dataset we are able to model this key component, and trace its effects on the AL, the MB, and learning. (iii) We tether our model architecture to a particular insect system (the *M. sexta* moth). As part of this tethering, (iv) we calibrate the model's AL firing rate behavior to a dataset of *in vivo* neural recordings of moths during learning, i.e., while exposed to both odors and octopamine. Thus our model is built “bottom-up,” with parameters as far as possible determined by a particular organism. For example, we set AL → MB connectivity and weights based on clues in biophysical studies and calibration to *in vivo* firing rates, rather than using the model to explore theoretically optimal values. This is an opposite, and complementary, approach to the studies cited above, and in combination with our unique *in vivo* octopamine data yields in several new findings, as well as some findings that reinforce those of previous studies but from a different angle.

We thus create a full, end-to-end neural network model (hereafter “Network Model”) that demonstrates robust learning behavior while also tightly matching the structure and behavior of a particular biological system. This approach has three advantages: (i) we can meaningfully compare Network Model simulation output to experimental data in order to tune model parameters; (ii) findings from our simulation results can map back to the original biological system to offer meaningful biophysical insights; and (iii) Network Model simulations allow us to study how the various key elements in the moth's toolkit (e.g., AL, MB, octopamine, and Hebbian updates) interact to enable learning. We can thus derive bio-inspired insight into the mathematical framework that enables rapid and robust learning in neural nets. Specifically, our experiments elucidate mechanisms for fast learning from noisy data that rely on cascaded networks, sparsity, and Hebbian plasticity.

These mechanisms have potential applications to engineered neural nets (NNs). NNs have emerged as a dominant mathematical paradigm for characterizing neural processing and learning, honoring their inspiration in the Nobel prize winning work of Hubel and Wiesel on the primary visual cortex of cats (Hubel and Wiesel, 1962). These seminal experiments showed that networks of neurons were organized in hierarchical layers of cells for processing visual stimulus. The first mathematical model of a neural network, the *Neocognitron* in 1980 (Fukushima, 1980), had many of the characteristic features of today's deep neural networks (DNNs). However, many of the biological motifs listed above (for insect AL-MBs) are largely absent from engineered NNs, whose principles and building blocks are biologically implausible even as DNNs have achieved great success (LeCun, 2015; Schmidhuber, 2015). For example, the AL-MB interaction with octopamine, Hebbian plasticity, and sparsity operates in a fundamentally different manner than the backprop optimization used in DNNs, and it also succeeds at tasks (e.g., rapid learning) where DNNs struggle. These biological mechanisms thus represent a potential opportunity to expand the set of structural and algorithmic tools available for ML tasks. We seek to characterize an actionable set of biological elements, a “biological toolkit,” that can be assembled into complementary NN architectures or inserted into engineered NNs, and that are capable of rapid and robust learning from very few training samples, an ability common in biological NNs but challenging for today's DNNs.

**Figure 1:**
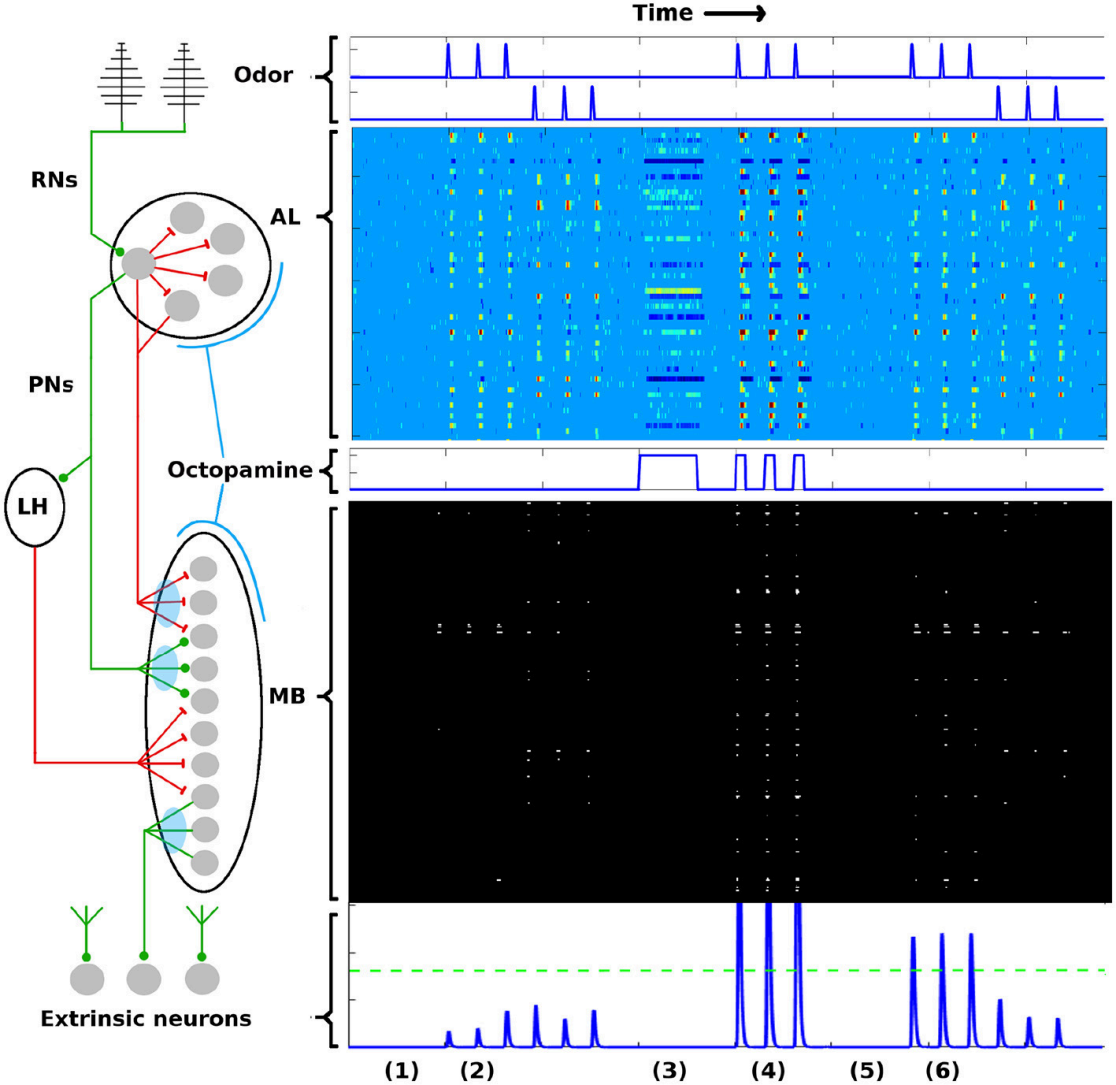
AL-MB overview. On the left is a system schematic: Chemical sensors (RNs) excite a noisy pre-amp network (AL), which feeds forward to a plastic sparse memory layer (MB), which excites readout (decision) neurons (ENs). Green lines show excitatory connections, red lines show inhibitory connections (LH inhibition of the MB is global). Light blue ovals show plastic synaptic connections into and out of the MB. On the right are neuron timecourse outputs from each network (typical simulation) with time axes aligned vertically. Timecourses are aligned horizontally with their regions-of-origin in the schematic. The AL timecourse shows all responses within ± 2.5 std dev of mean spontaneous rate as medium blue. Responses outside this envelope are yellow-red (excited) or dark blue (inhibited). MB responses are shown as binary (active/silent). Timecourse events are as follows: (1) A period of no stimulus. All regions are silent. (2) Two odor stimuli are delivered, 3 stimulations each. AL, MB, and ENs display odor-specific responses. (3) A period of control octopamine, i.e., without odor or Hebbian training. AL response is varied, MB and EN are silent. (4) The system is trained (octopamine injected) on the first odor. All regions respond strongly. (5) A period of no stimulus. All regions are silent, as in (1). (6) The stimuli are re-applied. The AL returns to its pre-trained activity since it is not plastic. In contrast, the MB and EN are now more responsive to the trained odor, while response to the untrained odor is unchanged. Green dotted line in the EN represents a hypothetical “action” threshold. The moth has learned to respond to the trained odor.

To briefly summarize the AL-MB network: It is organized as a feed-forward cascade of five distinct networks, as well as a reward mechanism (Hildebrand, 1996; Kvello et al., 2009; Martin et al., 2011). Roughly 30,000 noisy chemical receptor neurons (RNs) detect odor and send signals to the Antenna Lobe (AL) (Masse et al., 2009). The AL acts as a pre-amplifier, providing gain control and sharpening of odor representations (Bhandawat et al., 2007; Kuebler et al., 2012). It contains roughly 60 isolated units (glomeruli) (Huetteroth and Schachtner, 2005), each focused on a single odor stimuli feature (Christensen et al., 1995; Martin et al., 2011). Glomeruli laterally inhibit each other, and project odor codes to the Mushroom Body (MB). AL neurons are noisy (Lei et al., 2011; Galizia, 2014). The MB contains about 4,000 Kenyon Cells (KCs). These fire sparsely and encode odor signatures as memories (Campbell and Turner, 2010; Honegger et al., 2011; Balkenius and Hansson, 2012; Perisse et al., 2013). MB sparsity is enforced by global inhibition from the Lateral Horn (Bazhenov and Stopfer, 2010). Extrinsic Neurons (ENs), numbering ~10's, are believed to be “readout neurons” that interpret the KC codes (Campbell et al., 2013; Hige et al., 2015). In response to reward (sugar at the proboscis), a large neuron sprays octopamine globally over the AL and MB, causing generalized stimulation of neurons (Dacks et al., 2008; Riffell et al., 2012). Learning does not occur without this octopamine input (Hammer and Menzel, 1995, 1998). The connections into the KCs (AL → KCs) and out of the KCs (KCs → ENs) are plastic during learning (Cassenaer and Laurent, 2007; Masse et al., 2009). Figure 1 gives a system schematic (A) along with typical firing rate (FR) timecourses (from simulation) for neurons in each network (B). More network details are given in section 4.

## 2. Results

We first show the calibration of our Network Model to *in vivo* data. We then describe neural behaviors of the Network Model during learning and give results of learning experiments. Finally, we give results of experiments on MB sparsity.

### 2.1. Calibration of Model

The Network Model was calibrated to behave in a statistically similar way to three sets of *in vivo* data measuring projection neuron (PN) firing rate (FR) activity in the AL (see section 4 for details): (i) PN spike counts with odor but without octopamine: 129 units with FR>1 spike/sec, (ii) PN spike counts with odor and with octopamine: 180 units with FR>1 spike/sec, and (iii) PN spike counts with odor, with and without octopamine: 52 units with FR>1 spike/sec.

**Figure 2:**
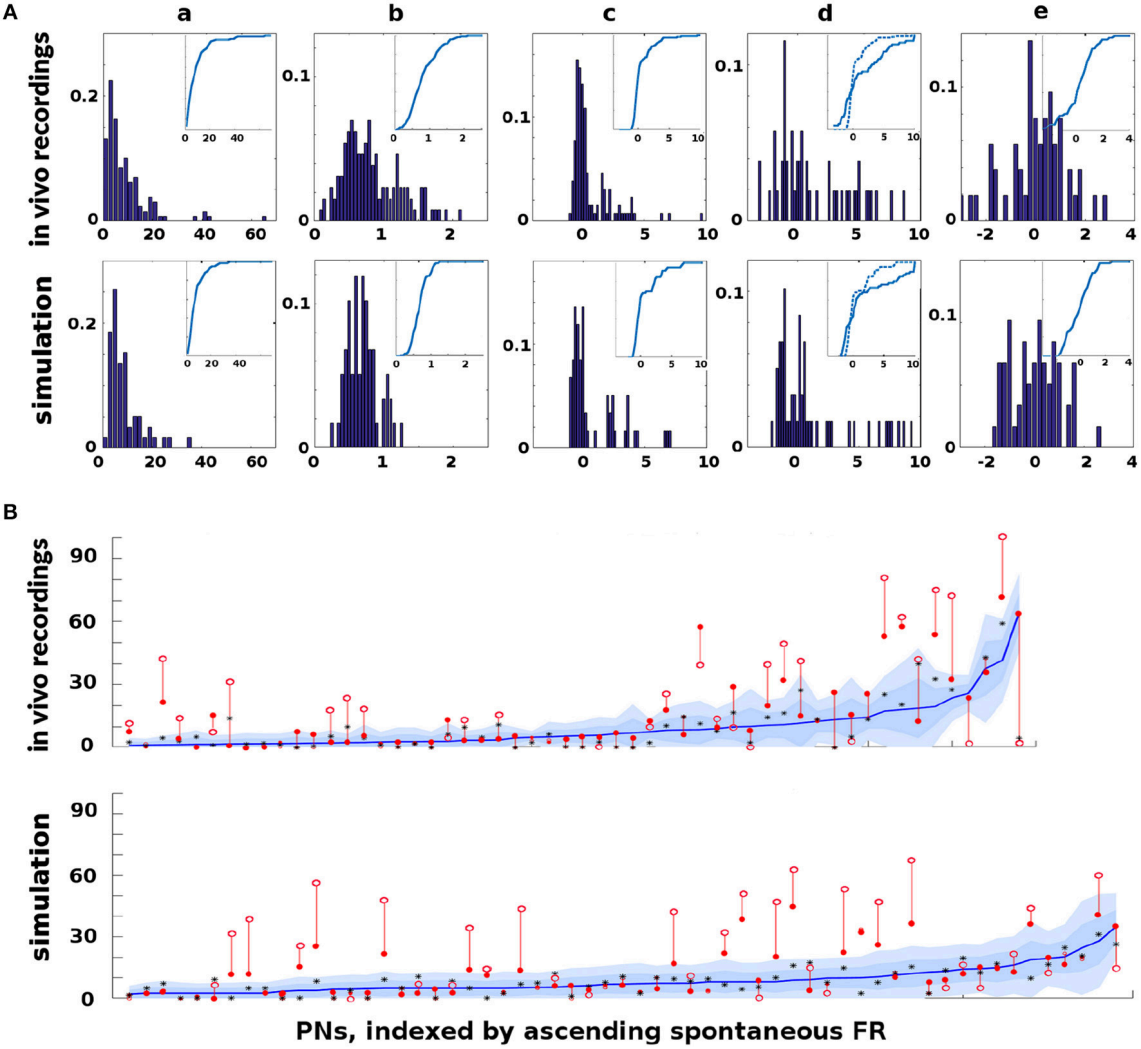
*In vivo* firing rate data and model calibration: Comparison of PN firing rate activity from *in vivo* data and simulations. **(A)** Histograms and CDFs of *in vivo* data and simulations. **(Col a)** Mean spontaneous FRs *μ*_*s*_. **(Col b)** *σ*_*s*_/*μ*_*s*_ of spontaneous FRs, a measure of noisiness of a PN. **(Col c)** Odor response, measured as distance from *μ*_*s*_ in *σ*_*s*_ units. Distance >2*σ*_*s*_ implies a strong activation/inhibition. **(Col d)** Odor response during octopamine, in *σ*_*s*_ units distance from *μ*_*s*_. Note that PN responses are broadened (i.e., more PNs are strongly activated or inhibited). The dotted line in the CDF inset is the same as the CDF of the odor response without octopamine, to show the broadening toward both extremes. **(Col e)** Change in mean spontaneous FRs due to octopamine, measured in *σ*_*s*_ units distance from (non-octopamine) *μ*_*s*_. Some PNs are excited, some are inhibited. **(B)** Activity of PNs indexed by increasing spontaneous FR. Blue lines = mean spontaneous FRs *μ*_*s*_ (cf col a). Shaded regions = *σ*_*s*_, 2*σ*_*s*_ envelopes (cf col b). Solid red dots = odor response FRs (cf col c). Hollow red dots = odor response FRs during octopamine (cf col d). Red lines show the change in odor response FRs due to octopamine (cf broadened response). Black stars (*) = spontaneous FRs during octopamine (cf col e). In **(A)** cols c, d, e, the x-axes are expressed in units of *σ*_*s*_, while in **(B)** the y-axis measures raw spikes/sec FR.

Due to the limited number of experimental units, only qualitative comparisons of the model and experiment could be made: Excessive tuning of the model parameters would have served only to overfit the particular data, rather than matching true PN behavior distributions or, more importantly, the general learning behavior of the moth. Figure 2 shows the close match of typical Network Model PN statistics to *in vivo* PN statistics based on mean (*μ*) and variance (*σ*) of spontaneous FRs and odor responses, both without and with octopamine (details of metrics are given in section 4). Importantly, Figure 2 shows significant octopamine-modulated increase in PN FR activity in the Network Model, consistent with *in vivo* experiments involving octopamine stimulation.

There is limited experimental data measuring the FR activity of Kenyon cells (KC) in the MB, and no data to our knowledge measuring KC in response to octopamine stimulation. However, we note that the behavior of KCs during the application of octopamine to the AL, either with or without odor, is not an artifact of parameter tuning. Rather, it follows from the tuning the AL to match *in vivo* data. Specifically, PN FRs at baseline (with no odor or octopamine), with odor alone, with octopamine alone, and with odor and octopamine, are all determined by calibration of the model to *in vivo* data. KCs respond only to PNs and to inhibition from the LH (see Figure 1). Calibrating the KC baseline response in the absence of octopamine to *in vivo* data in Turner et al. (2008) fixes the feed-forward connections from PNs. Assumed in this model, due to lack of biophysical evidence, is that octopamine has no direct stimulative effect on KC FRs (we do posit that it acts as an “on switch” for plasticity). Thus KC behavior with octopamine is fully determined once the model is tuned to PN data. This completes the calibration process of our model parameters. As Figure 2 shows, the model agrees well with *in vivo* experiment.

There are no bulk data, to our knowledge, measuring EN firing rates in response to odors and/or octopamine. However, calibrating EN response is not necessary to demonstrate an ability to learn. The key marker is post-training increase in EN response.

### 2.2. Learning Experiments: PN and KC Behaviors

PN activity in the AL, and KC activity in the MB, from typical Network Model simulations are shown in Figure 1 as heatmaps, evolved over the time course of a simulation in which the system was exposed to two different odors and trained on one of them. The AL is stimulated with octopamine during training. Each row of the heatmap represents a distinct PN or KC as it evolves in time (left to right columns of heat map). All the timescales are aligned. Neural behaviors are as follows:

#### 2.2.1. PNs

In the AL heatmap, the light blue region corresponds to PN FRs within 2.5 *σ*_*s*_ of their respective mean spontaneous FRs *μ*_*s*_, warm colors correspond to very high FRs, and dark blues correspond to strongly inhibited FRs. The simulations demonstrate a number of key PN behaviors, including (i) absent odor, PN FRs stay within their noise envelopes (by definition), (ii) the two odors have distinct excitation/inhibition signatures on PNs, (iii) octopamine alone (without odor) results in more PNs being excited beyond their usual noise envelopes, and also results in some PNs being inhibited below their usual envelopes, (iv) octopamine and odor, applied together, result in an overall excitation of PNs, and (v) the AL behavior returns to baseline after octopamine is withdrawn, since AL connection weights do not have (long-term) plasticity (Davis, 2005).

#### 2.2.2. KCs

In the MB, the KCs fire sparsely due to global inhibition from the Lateral Horn. The only plastic connections in the AL-MB system involve the KCs: Between PNs and KCs (*M*^*PK*^, *M*^*QK*^ in section 4); and between KCs and extrinsic readout neurons (ENs) (*M*^*KE*^ in section 4). Thus the KC odor signatures are modulated with training. Each row in the MB heatmap represents one of 500 randomly selected KCs in the simulation as it evolves in time (left to right columns of heat map). Black regions indicate FRs < 1 spike/sec, white regions indicate FRs > 1 spike/sec. The white regions have been dilated to make the sparsely-firing KCs easier to see.

The simulations demonstrate a number of key KC behaviors, including (i) the baseline KC FR response absent any odor is essentially zero, (ii) the two odors excite distinct sets of KCs with varying consistency from noise trial to noise trial, (iii) for a given odor, some KCs fire reliably in response to an odor stimulation and some fire only occasionally, (iv) when subject to octopamine but no odor, KCs are unresponsive, a benefit during learning since it prevents environmental noise from being encoded as meaningful, (v) when subject to both octopamine and odor, KCs respond strongly to the odor with high trial-to-trial consistency, and (vi) the global inhibition from the LH controls the level of sparseness in the KCs, both their silence absent any odor (with or without octopamine), and their sparse firing in response to odors.

**Figure 3:**
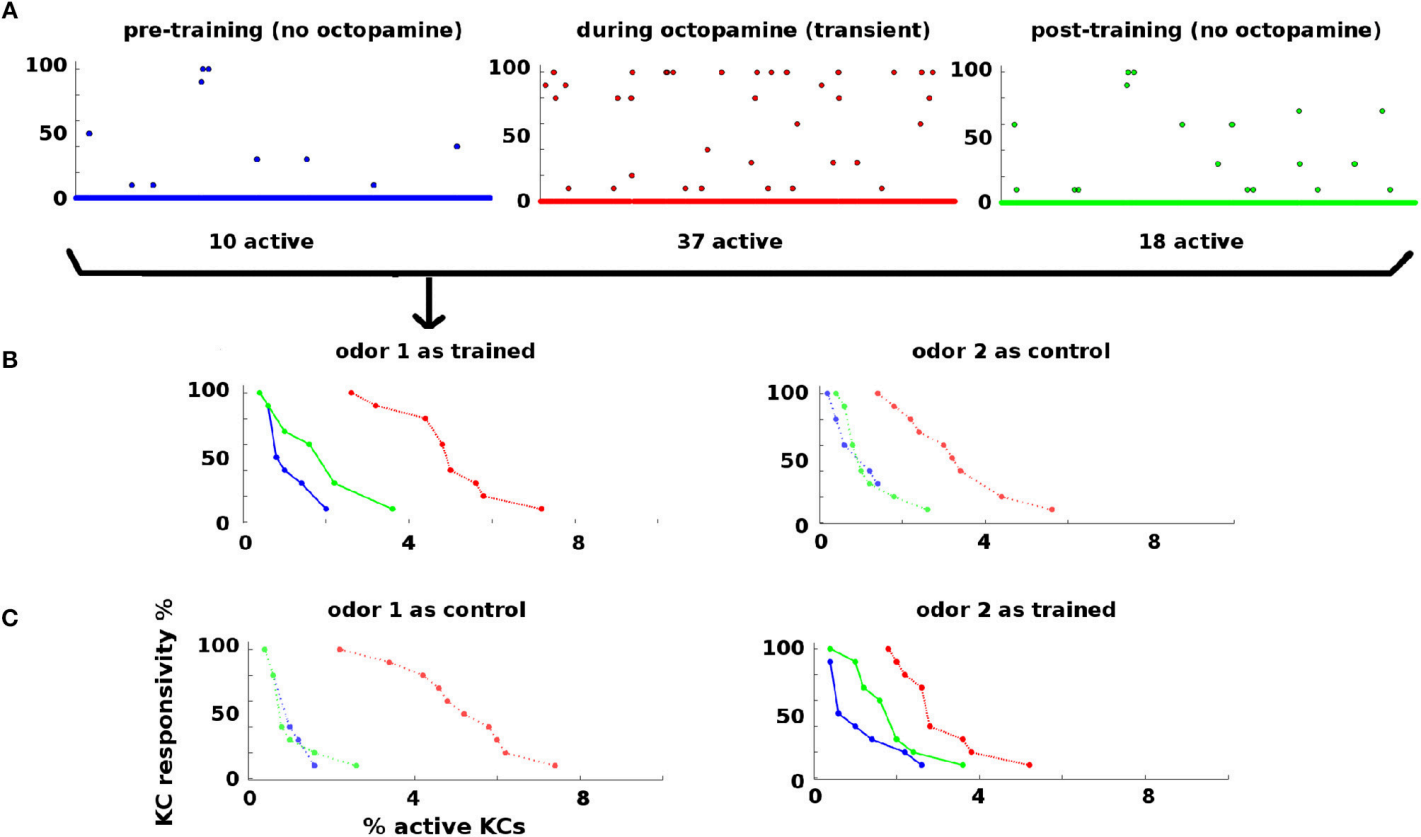
KC responses to odor during training: KCs respond sparsely to odor pre- and post-training, i.e., absent octopamine (blue and green dots and curves). Octopamine induces transient increased responsivity (red dots and curves). Training results in permanent increases in response to the trained odor, but no increase in response to control odor (green dots and curves). **(A)** KC response to an odor before, during, and after training. x-axis: indexed KCs (500 shown). y-axis: consistency of response (in %). The plots are for odor 1 as the trained odor (i.e., same data as **B**). Blue = pre-training (no octopamine). Red = during training (with octopamine); note the heightened transient response. Green = post-training (no octopamine). There is a permanent increase in the number of KCs that respond to the trained odor. **(B)** Response rate vs. percentage of active KCs for trained and control odors before, during, and after training. x-axis: percentage of KCs responding at the given rate. y-axis: consistency of response (in %). Blue, pre-training; Red, during octopamine (transient); Green, post-training. The LH plot shows odor 1 as the reinforced odor. The scatterplots in **(A)** correspond to the three curves in this plot. Note that the permanent KC response curve shifts up and to the right (blue → green) in the trained odor, i.e., more KCs respond to the odor (right shift) and they respond more consistently (upward shift). The RH plot shows odor 2 as a control. The control's permanent KC response curve does not shift. **(C)** As **(B)** above, but in this experiment odor 1 is now the control (LH plot), and odor 2 is reinforced (RH plot). In this case, the response curve of odor 2 (reinforced) shifts to the right (blue → green), while the response curve of odor 1 (control) is unchanged.

Statistics of KC responses to odors pre-, during, and post-training are shown in Figure 3. Naive moths have low KC response to odors, in both percentage of KCs activated and their consistency of response to odor stimulations (Figure 3, blue dots and curves). During training octopamine induces high KC response, in both percentage and consistency (Figure 3, red dots and curves). After octopamine is withdrawn, KC response is lower than during training, but remains higher than naive levels in both percentage and consistency (Figure 3, green dots and curves) for the trained odor only. Thus the newly-learned importance of the trained odor is encoded as broader and stronger KC responses by means of strengthened synaptic connections.

EN (readout neuron) activity is also shown over time at the bottom of Figure 1. Learning is evidenced by the increased EN response to the trained odor even after octopamine has been withdrawn, due to Hebbian growth of synaptic connections into and out of the MB.

The FR activity of the PNs in the AL, the KCs in the MB, and the ENs, as illustrated in Figures 1, 3, demonstrate the entire learning process that occurs under the influence of octopamine stimulation. Without octopamine, learning does not occur.

Interestingly, although the AL does not itself experience plasticity changes, it is the AL's increased FR activity (induced by octopamine) which enables permanent synaptic weight changes in the MB via Hebbian plastic updates.

### 2.3. Learning Experiments: EN Behavior

A key finding of this paper is that the AL-MB model demonstrates robust learning behavior. Here “learning” is defined as permanently modifying synaptic weights in the system so that the reinforced odor yields a significantly stronger response in the readout neuron (EN) post-training, relative to naive (i.e., pre-training) response to that odor, and also relative to the post-training responses to control odors.

#### 2.3.1. Structure of Learning Experiments

Moths were randomly generated from a fixed parameter template, which included randomly-assigned input maps (odor → AL) for four odors. The odors projected broadly onto the AL, each odor hitting ~20 out of 60 glomeruli. As a result, their projections onto the AL overlapped substantially. A combinatorial calculation (4 independent draws of “60 choose 20”) shows that, on average, a given odor projected uniquely onto about 6 glomeruli, and shared its other 14 glomeruli with other odors. Each generated moth was put through a series of training experiments, with each run in the series structured as follows:

1. The moth first received a series of stimulations from each odor, to establish a baseline (naive) EN response. The stimulations were 0.2 s long and separated by gaps of several seconds.
2. The moth was trained on one of the odors for 1–4 sessions (one session = 5 odor stimulations), by applying odor and octopamine concurrently. The MB plastic weights were updated according to a Hebbian rule.
3. Upon completion of training, the four odors were each again applied as a series of odor stimulations, to establish post-training EN response.

For each {odor, #sessions} pair, this experiment was conducted 11 times (i.e., 11 noise realizations), for a total of 176 experiments on each moth. These results were aggregated to assess the particular moth's learning response.

#### 2.3.2. Learning Experiment Results

**Figure 4:**
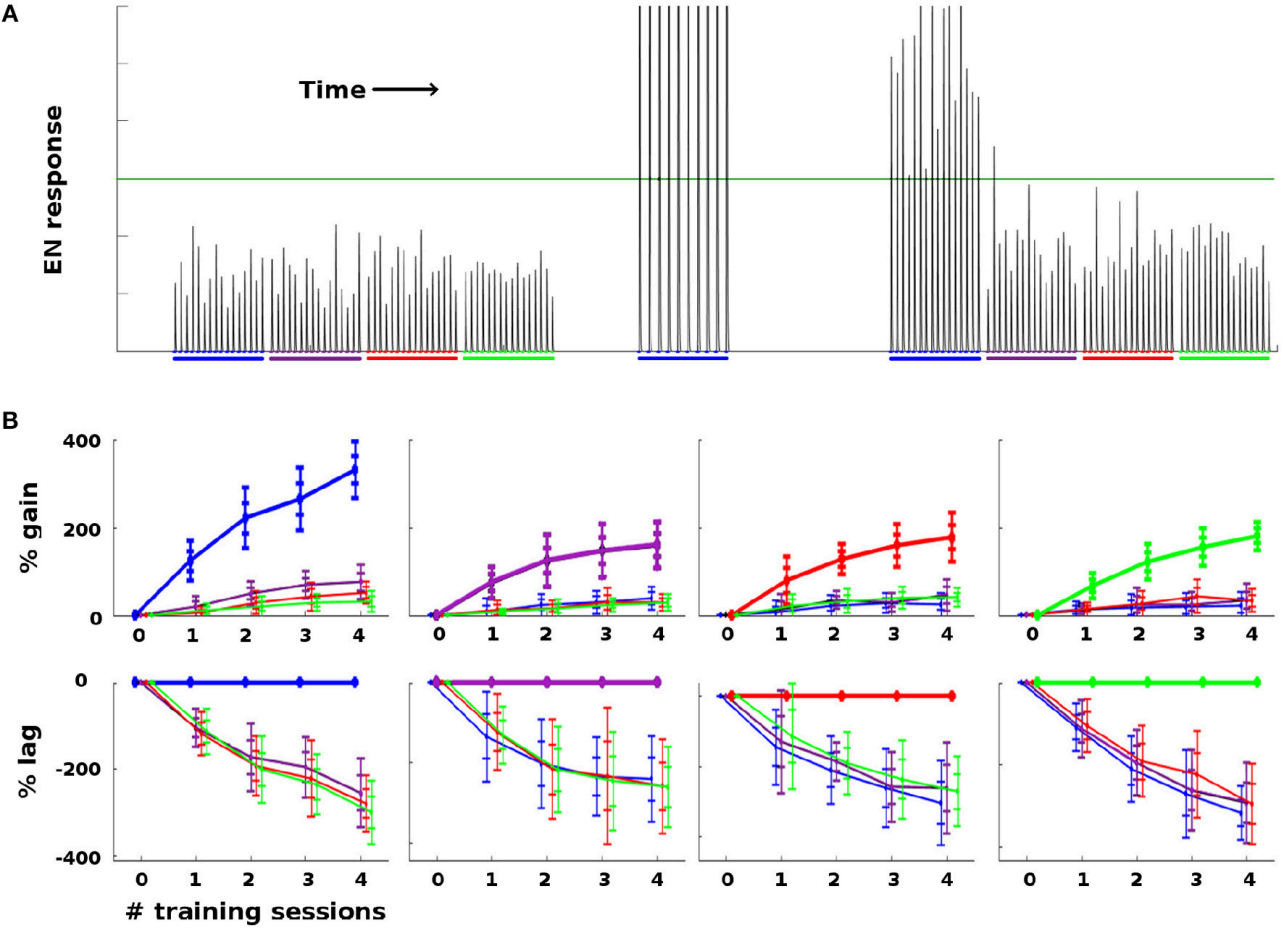
Effect of training on EN FRs: **(A)** Typical timecourse of EN responses from an experiment with a single moth. First, 16 stimulations of each odor were delivered, to establish naive odor responses. Note EN response variability due to noise in the system, especially in the AL. Next, the moth was trained on the first (blue) odor trained over 2 sessions (10 stimulations), by delivering odor and octopamine concurrently. This timecourse corresponds to the {odor, #sessions} pair in the first column in **(B)**, at index 2 on the x-axis. Octopamine was then withdrawn, and the four odors were again delivered in series of stimulations, to establish post-training changes in EN response. The long green line represents a hypothetical trigger threshold, such that EN response > threshold would induce a distinct behavior. **(B)** EN response changes due to training, aggregated results with 11 noise realizations for each {odor, #sessions} pair. Each column shows results of training a given odor, color coded: blue, purple, red, green. x-axis = number of training sessions. First row: The y-axis measures percent change in EN FR. The line shows mean percent change. The error bars show ±1, 2 stds. Second row: The y-axis measures percent changes in EN response, relative to the trained odor (i.e., subtracting the trained odor's change from all odors). This shows how far each control odor lags behind the trained odor. The line shows mean percent lag. The error bars show ±1, 2 stds.

As a general rule, Network Model moths consistently demonstrated strong learning behavior in terms of EN response: Training increased EN response to the trained odor well beyond naive levels, and also much more than it affected EN response to control odors. Figure 4 summarizes the changes in EN responses in a typical experiment on a moth with four odors. Figure 4A shows a typical noise realization timecourse, where one odor was reinforced with octopamine and the other three odors were controls. Figure 4B shows the statistics of EN response modulation, according to {odor, #sessions} pairs.

For ease of interpretation, the moth shown in Figure 4 had naive EN responses of roughly equal magnitude for all four odors. When naive EN response magnitudes were highly uneven (> 3x), robust learning still occurred, but the interpretation of the results is more complex due to scaling issues. A typical experiment using a moth with odor responses of highly unequal magnitude is shown in Supplementary Material.

#### 2.3.3. Points of Interest (EN Responses to Learning)

1. Because EN response is driven solely by feed-forward signals from KCs, ENs had response ≈ 0 in the absence of odor, with or without octopamine, as expected (since KCs are silent absent any odor). Thus Hebbian growth during training did not increase EN baseline (no-odor) response.
2. The EN response to odor + octopamine was always very strong, as seen in Figure 4A, where EN responses to odor + octopamine extend above the top of the figure. Note that this effect follows automatically from the calibration of the Network Model to *in vivo* data. Its functional value to the moth is addressed in the section 3.
3. Training consistently increased the EN response to the reinforced odor much more than EN response to control odors, measured as percentage of naive odor response.

Since the Network Model did not include a Hebbian decay dynamic (for simplicity, absent clear evidence), this was the key indicator of robust learning. That is, focused learning was expressed by substantially higher increase in EN response to reinforced vs. control odors. We assume that an added Hebbian decay term would have knocked smaller increases back, thus returning control odor responses to baseline.

Results of ANOVA analysis for differential effects of training on reinforced vs unreinforced odors shows that when naive odor EN response magnitudes were similar (within 3x of each other) *p*-values were consistently < 0.01. ANOVA analysis results are given in Supplementary Material.

### 2.4. MB Sparsity Experiments

Projection into a high-dimensional, sparse layer is a common motif in biological neural systems (Ganguli and Sompolinsky, 2012; Litwin-Kumar et al., 2017). To explore the role of MB sparsity during learning, we ran Network Model experiments that varied the level of generalized inhibition imposed on the MB (the lateral horn, LH, controls MB sparsity level). Each experiment set a certain level of LH inhibition, then ran simulations (see section 4) that trained moths on one odor with 15 odor stimulations and left one control odor untrained. EN responses to both trained and control odors were recorded, as well as the percentage of KCs active in response to odor.

Too little damping from the LH resulted in a high percentage of KCs being active (low sparsity). This regime gave consistent EN responses to odor. But it also caused EN responses to both control odor and noise to increase significantly during training, reducing the contrast between EN responses to trained and control odors and also increasing spontaneous EN noise.

Too much damping resulted in a very low percentage of KCs being active (high sparsity). This ensured that training gains were focused on the trained odor while EN response to control odors and noise were not boosted. However, in this regime EN responses to all odors, both pre- and post-training, were generally unreliable because too few KCs were activated.

**Figure 5:**
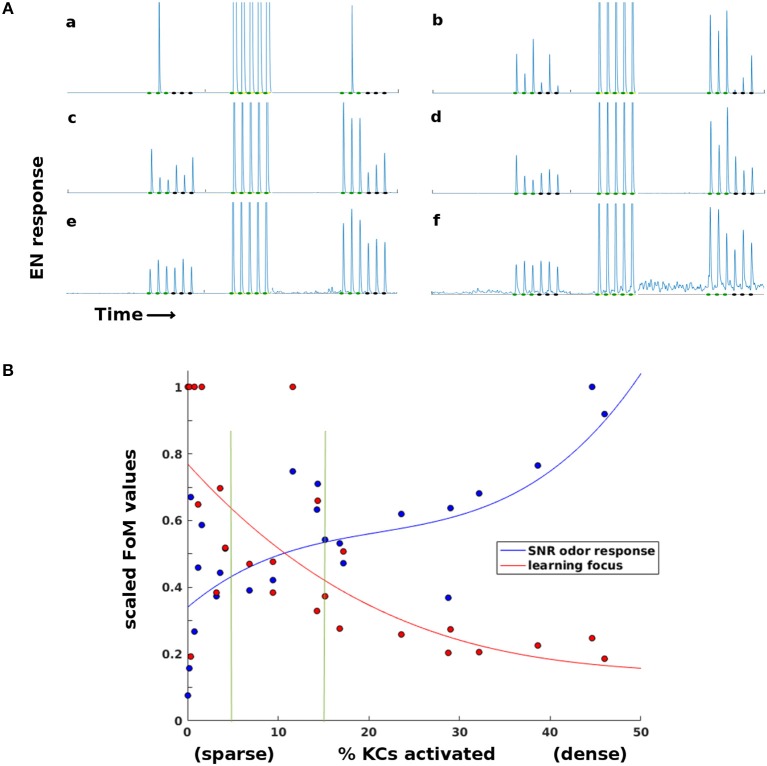
Effects of sparsity on learning and EN reliability. Results for a typical experiment on a moth with two odors. **(A)** EN responses timecourses for two odors, at varying levels of KC activation (a,b: < 1%. c,d: 5–15%. e,f: 20–45%. Order of events: three stimulations of each odor as baseline, train on first odor (only one session shown), then three stimulations each post-training. At very sparse levels (a,b) training is focused but odor response is not reliable. At low sparsity levels (e,f) training is unfocused, boosting EN response to control odor and to background noise. **(B)** Two Figures of Merit (FoMs) plotted against MB sparsity. Low KC activation (high sparsity) correlates with well-focused learning, but low odor response SNR. High KC activation (low sparsity) correlates with poorly-focused learning, but high odor response SNR. The FoMs are each normalized for easier plotting. y-axis: Blue data: $\frac{\mu \left(f\right)}{\sigma \left(f\right)}$, a measure of odor EN response SNR, where *f* = EN odor response. Red data: $\frac{\mu \left(f_{T}\right)}{\mu \left(f_{C}\right)}$, a measure of learning focus, where *μ*(*f*_*T*_) = mean EN post-training response to reinforced odor; *μ*(*f*_*C*_) = mean EN post-training response to control odor (values are thresholded at 1 for plotting). A high value indicates that increases in EN response due to training were focused on the trained odor; low values indicate that irrelevant signal (*F*_*C*_) was also boosted by training. The points are experimental data, the curves are cubic fits. Vertical green lines indicate the 5–15% sparsity region, typical in biological neural systems.

Thus sparseness in the high-dimensional MB fulfilled a vital role in the Network Model's learning system. LH inhibition of the MB had an optimal sparsity regime that balanced opposing demands: KC firing had to be sufficiently dense for reliable odor response on one hand, and sufficiently sparse for well-targeted Hebbian growth on the other. Timecourses illustrating the effects of too-little or too-much sparsity are seen in Figure 5A. Figure 5B shows how this trade-off varied with MB sparsity, by plotting two figures-of-merit:

(1) $$\begin{array}{rcl} \text{Signal-to-Noise Ratio (SNR)} & = & \frac{\mu \left(f\right)}{\sigma \left(f\right)},\text{where} \end{array}$$

*f* = EN odor response.

(2) $$\begin{array}{r} \text{“Learning Focus”}=\frac{\mu \left(f_{T}\right)}{\mu \left(f_{C}\right)}\text{, where} \end{array}$$

*μ*(*f*_*T*_) = mean post-training EN response to trained odor, *μ*(*f*_*C*_) = mean post-training EN response to control odor.

## 3. Discussion

Because we took a distinct approach to designing our Network Model, and because we had access to unique *in vivo* octopamine data, our experiments yield novel insights into the moth olfactory network and how it learns. This discussion focuses on four areas: (i) predictions about aspects of the AL-MB still unclear in the literature, (ii) the role of sparse layers, (iii) the role of octopamine, and (iv) the value of noise. In addition, we consider these insights in the context of Machine Learning.

### 3.1. Predictions Re Details of AL-MB Structure

Because our Network Model in tethered to a particular system, both the calibration process and simulations offer hints as to some unresolved biophysical aspects of the moth's AL-MB system. Some examples:

#### 3.1.1. Do LNs Inhibit PNs and LNs as Well as RNs

In the AL, LNs have a net inhibitory effect on PNs (Lei et al., 2002; Olsen et al., 2010), but the exact means to this end are not clear. In particular, while LNs are known to inhibit RNs (Olsen et al., 2010), it is less clear whether or to what degree LNs also directly inhibit PNs and LNs. Efforts to calibrate our Network Model to *in vivo* data indicate that LNs need to inhibit not just RNs, but also (to a lesser degree) LNs and PNs. The model weight strengths for LN → RN, → LN, and → PN are in the ratio of 6:2:1. That LNs would inhibit LNs makes sense when the goal is maximizing PN output of the active glomerulus: By inhibiting the LNs of rival glomeruli, the active glomerulus reduces the amount of inhibition directed at itself. Similarly, that LNs would inhibit PNs makes sense if the goal is to reduce the PN output of rival glomeruli.

#### 3.1.2. Octopamine's Effects on Different Neuron Types

Octopamine increases the responsivity of a neuron to incoming signals. It is unclear how or whether octopamine affects various neuron types (i.e., RNs, PNs, LNs, KCs). Calibration of the Network Model's AL behavior, and tuning of KC behavior to enable learning, indicate that octopamine needs to target RNs and LNs, but not PNs, KCs, or ENs. Logical arguments support these findings:

**RNs:** Because RNs initially receive the odor signal, these are logical neurons to stimulate with octopamine, because it sharpens their response to the exact signature being trained, which in turn sharpen the AL's output code for that odor.

**LNs:** LNs have the dual roles of inhibiting rival glomeruli and limiting overall PN output in the AL. For the first role, increased LN response to RNs will tend to sharpen AL response to the trained odor, by accentuating inhibition of rival glomeruli PNs. For the second role, increased LN activity mitigates the risk that increased RN activity (due to octopamine) might blow up the overall PN output of the AL.

**PNs:** Our Network Model simulations suggest that PNs should receive little or no octopamine stimulation. While increasing PN responsivity would benefit RN-induced sharpening of the trained odor's signature, there are three downsides. First, RN input to PNs is intrinsically noisy, so higher PN responsivity amplifies noise as well as signal. Second, since PNs respond to LNs, higher PN activity tends to reduce the impact of LN inhibition, and thus reduces the inhibition-induced sharpening of the AL odor response caused by octopamine. Third, increasing PN responsivity can have an outsize effect on overall PN firing rates, i.e., it is a “high-gain” knob and therefore risky.

**KCs:** Our Network Model simulations indicate that direct octopamine stimulation of KCs greatly reduces sparseness in the MB (given the mechanics of our global inhibition on KCs), which can be disastrous to learning. Thus we expect that octopamine has no, or only slight, direct stimulative effect on KCs. However, other forms of global inhibition might admit direct octopamine stimulation of KCs while still preserving sparsity: (i) If the strength of the Lateral Horn's inhibition signal tracks the AL output, then octopamine stimulation of KCs would be offset by increased inhibition from the LH, due to increased AL output to the LH, preserving sparsity; (ii) if the KC population generates the inhibition signal, as in Lin et al. (2014), then octopamine stimulation of KCs would result in a counteractive stronger inhibition, again preserving sparsity. The arguments for why PNs should receive very little direct octopamine stimulation (given above) apply to KCs as well; but it is also possible that direct stimulation of KCs might improve learning by enabling random exploration of the odor-coding solution space (as mooted below for octopamine).

### 3.2. Noise Filtering Role of the Sparse, High-Dimensional Stage

Projection from a dense, low-dimensional coding space (eg the AL) to a sparse, high-dimensional coding space (e.g., KCs in the MB) is a widespread motif of biological neural systems, with size shifts routinely on the order of 20x–100x (Ganguli and Sompolinsky, 2012; Babadi and Sompolinsky, 2014; Litwin-Kumar et al., 2017). Some proposed reasons include information capacity, long-range brain communication, and reduced training data needs (Ganguli and Sompolinsky, 2012), as well as better inherent discrimination ability (Bazhenov et al., 2013; Litwin-Kumar et al., 2017; Peng and Chittka, 2017).

Our Network Model experiments highlight another key role of sparseness, relevant to learning: It acts as a robust noise filter that prevents the Hebbian growth process from amplifying upstream noise to out-of-control levels. Though noise may be useful (or unavoidable) in upstream networks such as the AL, noise that reaches the neurons on both sides of a plastic synaptic connection will be amplified by Hebbian growth during learning, swamping the system's downstream neurons (e.g., ENs) with noise.

However, the “fire together, wire together” principle of Hebbian learning is an AND gate. Thus it suffices to remove noise from just one of the two connected neurons to eliminate synaptic growth. Sparsity does precisely this, and is arguably a necessary part of a workable Hebbian learning mechanism. We also find that high sparsity focuses learning (even absent upstream noise), i.e., it enables better learned separation of classes, in agreement with (Huerta and Nowotny, 2009; Peng and Chittka, 2017). The negative effect of high sparsity on SNR (Figure 5) found in our experiments meshes with a similar observation in Nowotny (2009).

Setting aside the particular demands of Hebbian plasticity, robust noise filtering may be a core function of sparse, high-dimensional stages within any network cascade where noise accumulates due to (beneficial) use in upstream stages.

### 3.3. Roles of Octopamine

The levels of octopamine stimulation in our Network Model were calibrated to *in vivo* data on PN responses to octopamine. Thus, our simulations give novel insights into downstream effects of octopamine on plasticity, KC responses, EN responses, and Hebbian learning.

#### 3.3.1. Accelerant

Moths can learn to respond to new odors remarkably quickly, in just a few exposures. Our simulations indicates that while Hebbian growth can occur without octopamine, it is so slow that actionable learning, i.e., in terms of amplified EN responses, does not occur.

This implies that octopamine, through its stimulative effect, acts as a powerful accelerant to learning. Perhaps it is a mechanism that allows the moth to work around intrinsic organic constraints on Hebbian growth of new synapses, constraints which would otherwise restrict the moth to an unacceptably slow learning rate. To the degree that octopamine enabled a moth to learn more quickly, with fewer training samples, it would clearly be highly adaptive.

#### 3.3.2. Active Learning

Our simulations indicate that octopamine strongly stimulates the EN response to even an unfamiliar odor. Since octopamine is delivered as a reward, this has a beneficial effect in the context of reinforcement learning (Sutton and Barto, 1998), with the moth as the learning agent. An agent (the moth) can in some cases learn more quickly when it has choice as to the sequence of training samples (Active Learning, Settles, 2012).

In particular, when a certain class of training sample is relatively rare, it benefits the agent to actively seek out more samples of that class (Attenberg and Provost, 2010). Octopamine enforces high EN response to a reinforced odor, ensuring that ENs will consistently exceed their “take action” threshold during training. If the action is to “approach,” the moth is more likely to again encounter the odor, thus reaping the benefits predicted by Active Learning theory. This advantage applies in the context of positively-reinforced odors.

In the case of aversive learning, the high EN responses to unfamiliar but objectionable odors, due to dopamine, would cause the moth to preferentially avoid further examples of the odor. This would slow learning of aversive responses (a drawback), but would also minimize the moth's exposure to bad odors (danger avoidance, a benefit).

#### 3.3.3. Exploration of Optimization Space

A limitation of Hebbian growth is that it can only reinforce what already exists. That is, it only strengthens channels that are transmitting signals deemed (by association) relevant to the stimulus being reinforced. Absent a mechanism like octopamine, this constrains growth to channels that are already active. Our simulations indicate that octopamine induces much broader activity, both upstream from and within the plastic layer, thus activating new transmitting channels. This allows the system to strengthen, and bring permanently online, synaptic connections that were formerly silent. This expands the solution space the system can explore during learning. This function may be particularly important given the constraint of sparsity placed on odor codes in the MB.

#### 3.3.4. Injury Compensation

There is evidence that many forms of injury to neurons result in dropped spikes and thus lower firing rates in response to odors (Maia and Kutz, 2017). This injury-induced drop in the signals reaching the ENs could induce behavioral consequences, by lowering EN responses to below key behavioral action thresholds. Experiments in Delahunt et al. (2018) suggest that octopamine drives a mechanism to compensate for this type of neural injury.

Suppose that injury has reduced an upstream neural signal, such that a downstream EN can no longer exceed its behavioral action threshold. Octopamine stimulation of the upstream network will temporarily boost the reduced (injured) signal strength, so that the input signals to the ENs are above threshold during training. This in turn allows Hebbian growth to strengthen the synaptic connections to those ENs. Once octopamine is withdrawn, the inputs from the (still-injured) upstream network revert to their reduced level. But due to the newly-strengthened connection weights, these reduced inputs suffice to push EN response above its action threshold, restoring EN-controlled behaviors to their pre-injury baseline.

This mechanism (octopamine stimulation plus Hebbian synaptic growth) might allow neural systems to regain behavioral function lost due to damage to upstream regions, by increasing connection strengths downstream from the point of injury. Indeed, given the vital importance of injury mitigation to survival, it is possible that the “learning” mechanism originally evolved for the purpose of restoring behavioral function impaired by neural damage.

### 3.4. The Value of Noise

Noise in biological neural systems is believed to add value (besides just being cheap), for example by encoding probability distributions and enabling neural Baysian estimations of posteriors (Ma et al., 2006). In addition, injection of noise while training engineered NNs can improve trained accuracy (An, 1996). Our experiments indicate that in the AL-MB, noise has two other potential benefits, coupled with a caveat.

First, noise in the AL adds an extra dimension to MB odor encoding, increasing the granularity of its odor responses (Figure 3). The MB responds to odors in two ways: (i) by the number of KCs that are responsive, and (ii) by the reliability (eg from 10 to 100%) of their responses. This can be seen in the effect of octopamine on KC odor response, Figure 3B. Octopamine boosts MB odor response by increasing the number of active KCs (horizontal shift in response curves) and also by increasing the reliability of responsive KCs (vertical shift in responsivity curves). Both these shifts represent a stronger MB response and translate into stronger EN response.

Taken together, they provide a finer granularity of the response range than does the binary response of a noise-free system. That is, the MB response to noisy inputs from the AL is a concrete example of a mechanism used by a neural system to translate the probability distributions encoded by noisy neurons into actionable signals with high dynamic range and granularity.

Second, the system is also potentially robust to noisy stimuli. In the neural net context, input samples (i.e., inputs to the feature-reading layer) can be thought of as a *de facto* “first layer” of the neural net. A system that is robust to upstream noise may also be naturally robust to noisy inputs, a further potential advantage of judicially-placed sparse layers.

The caveat is that noise in the AL-MB must be confined to the AL, i.e., upstream from the encoding layer, in order to protect the readout neurons and Hebbian learning mechanism from noise. The system's success depends on robust noise filtering at the MB layer, via global inhibition from the LH. So the three-stage architecture consisting of: “Noisy pre-amplifier layer → Sparse noise-reduction layer → Decision layer” is an interdependent system well-suited to nuanced decision-making.

### 3.5. Applications to Machine Learning

The model and simulations in this paper characterize key features of the AL-MB system which might usefully be ported to machine learning algorithms. These features include: Generalized stimulation during training; Hebbian growth; sparse layers to control plastic connections and filter noise; and noisy initial layers. Advantages of this biological toolkit include:

#### 3.5.1. Fast Learning

Moths can reliably learn a new odor in less than 10 exposures, and biological brains in general can learn given very few training samples. This contrasts by orders of magnitude with the voracious data demands of DNNs, for which assembling sufficient training data can be a serious chokepoint in deployment. Indeed, when learning handwritten digits from very few samples (1–10 per class), an insect brain outperforms ML methods including CNNs Delahunt and Kutz (2018). These “fast and rough” biological mechanisms, seen in the moth in their simplest form, thus have potential to act as a complement to the precise but slow learning of DNNs.

#### 3.5.2. Robustness to Noise

The sparse layer in the AL-MB acts as an effective noise filter, protecting the readout neurons from a noisy upstream layer (the AL). Since the system is designed to accommodate upstream noise, it is possible that it can also readily accommodate noisy input samples. NNs have a troublesome property, that input-output score functions are not locally continuous (Szegedy et al., 2013). Biological neural nets seem to avoid this particular fault (or at least have different, complementary discontinuities). The noisy layer → sparse layer motif may be one reason for this. It may thus be a useful motif to apply in ML architectures.

#### 3.5.3. Novel Training Mechanism

Hebbian growth, combined with octopamine stimulation and the focusing effect of sparse layers, is a novel (in the context of ML) mechanism to explore a solution space and train a classifier. In particular, it works on a different principle than the backprop algorithm that drives DNNs: It does not minimize a loss function via gradient descent, nor does it punish incorrect answers; rather, it selectively strengthens those connections that transmit meaningful signals, and weakens connections that are inactive. We argue that this biological optimization mechanism is of potential value to ML because it is (i) functionally distinct from the backprop algorithm currently used, and (ii) known to succeed in regimes (e.g., rapid learning) where backprop struggles.

#### 3.5.4. Biological Plausibility

One characteristic (not criticism) of backprop is its biological implausibility, since it requires a neuron to have more than local knowledge of the system. A current area of interest, especially in the context of DNNs (Bengio and Fischer, 2015), is the search for neural network architectures (for example with recurrent connections to transport non-local information) and variants of backprop which are biologically plausible, which might narrow the gap between biological and engineered NNs. Our experiments demonstrate that the triad of octopamine stimulation + Hebbian growth + sparse layers can efficiently train a NN, and is thus a possible candidate to address the biological plausibility gap.

## 4. Materials and Methods

This section gives a detailed description of the biological moth olfactory network, as well as our Network Model. The biological detail is relevant because our model is built “from the ground up,” i.e., it is based on a particular organism, rather than being a “top-down,” more theoretically-based, architecture. We also provide a Glossary, and describe the *in vivo* data used for model calibration.

### 4.1. Moth Olfactory System Overview

The parts of the AL-MB implicated in learning are organized as a feed-forward cascade of five distinct networks, as well as a reward mechanism (Kvello et al., 2009; Martin et al., 2011). Figure 1 gives a system schematic along with typical firing rate (FR) timecourses (from simulation) for neurons in each network.

1. Antennae. Roughly 30,000 noisy chemical receptors detect odor and send signals to the Antenna Lobe (Masse et al., 2009).
2. Antenna Lobe (AL). Contains roughly 60 units (glomeruli), each focused on a single odor feature (Martin et al., 2011). The AL essentially acts as a pre-amp, boosting faint signals and denoising the antennae inputs (Bhandawat et al., 2007). AL neurons are noisy (Galizia, 2014).
3. Lateral Horn (LH). Though not fully understood, one key function is global inhibition of the Mushroom Body to enforce sparseness (Bazhenov and Stopfer, 2010).
4. Mushroom Body (MB), here synonymous with the Kenyon Cells (KCs). About 4,000 KCs are located in the calyx of the Mushroom Body (MB). These fire sparsely and are believed to encode odor signatures (Campbell and Turner, 2010; Honegger et al., 2011; Perisse et al., 2013). KCs are believed to be relatively noise-free (Perez-Orive et al., 2002).
5. Extrinsic Neurons (ENs), numbering ~10's, located downstream from the KCs. These are believed to be “readout neurons” that interpret the KC codes and convey actionable messages (such as “fly upwind”) (Campbell et al., 2013; Hige et al., 2015).
6. Reward Mechanism. A large neuron sprays octopamine globally over the AL and MB, in response to reward, such as sugar at the proboscis. Learning does not occur without this octopamine input (Hammer and Menzel, 1995, 1998). The neuromodulator dopamine works similarly, but drives aversive learning (Dacks et al., 2012).
7. Inter-network connections: In the AL-MB these are strictly feed-forward, either excitatory or inhibitory. In particular, Antennae → AL, AL → LH, KCs → ENs are all excitatory. LH → KCs is inhibitory. AL → KCs have both excitatory and inhibitory channels.
8. Plasticity: The connections into the KCs (AL → KCs) and out of the KCs (KCs → ENs) are known to be plastic during learning (Cassenaer and Laurent, 2007; Masse et al., 2009). The AL does not have long-term plasticity (Davis, 2005).

### 4.2. Component Networks and Their Network Model Representations

This subsection offers a more detailed discussion of the constituent networks in the biological AL-MB, and details about how they are modeled in our Network Model.

#### 4.2.1. Antennae and Receptor Neurons

The Antennae receptors, activated by chemical molecules in the air, send excitatory signals to Receptor Neurons (RNs) in the AL. Several thousand antennae converge onto 60 units (glomeruli) in the AL (Nagel and Wilson, 2011). All the receptors for a given atomic volatile converge onto the same glomerulus in the AL, so the glomeruli each have distinct odor response profiles (Deisig et al., 2006). Since natural odors are a blend of atomic volatiles, a natural odor stimulates several units within the AL (Riffell et al., 2009b).

Our model does not explicitly include antennae. Rather, the first layer of the model consists of the RNs entering the glomeruli. Though ~500 RNs feed a given glomerulus, the model assumes one RN. The benefit of many RNs converging appears to be noise reduction through averaging (Olsen et al., 2010). This can be simulated by one RN with a smaller noise envelope.

Each glomerulus' RN has a spontaneous FR and is excited, according to random weights, by odor stimuli.

#### 4.2.2. Antenna Lobe and Projection Neurons

**Figure 6:**
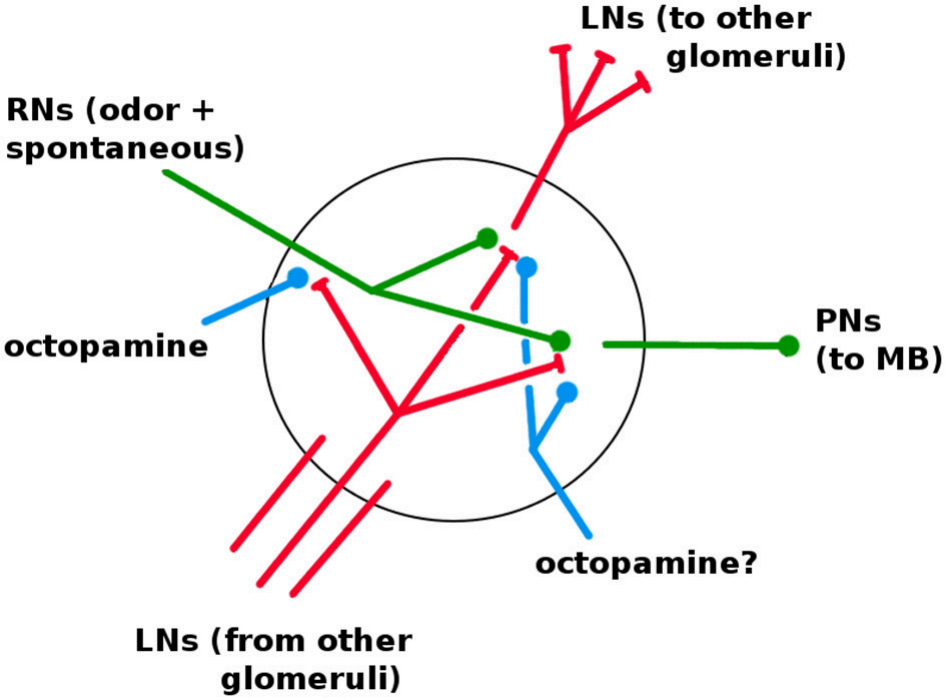
Detail of neural connections within a glomerulus. Red, inhibitory; green, excitatory; blue, increases responsiveness. RNs enter from the antennae. LNs enter from other glomeruli; one full LN is shown. It is not known if octopamine modulates LNs and PNs (see section 3.1).

The AL is fairly well characterized in both structure and dynamics, with a few important gaps. It contains about 60 glomeruli, each a distinct unit which receives RN input and projects to the KCs via excitatory PNs. The same PN signal also projects to the LH (Bazhenov and Stopfer, 2010). The AL, unique among the networks, has inhibitory lateral neurons (LNs) (Wilson and Laurent, 2005), the only neurons that are not strictly feed-forward. (There is some evidence of excitatory LNs, e.g., Olsen et al., 2008; the Network Model excludes this possibility.) The LNs act as a gain control on the AL, and also allow odors to mask each other by inhibiting other glomeruli's RNs (Olsen and Wilson, 2008; Hong and Wilson, 2015). It is not known whether LNs also inhibit PNs and LNs. Based on calibrations to *in vivo* data, in Network Model LNs inhibit all neuron types (cf section 3.1). Thus each glomerulus contains dendrites (i.e., outputs) for PNs and LNs, and axons (i.e., inputs) for RNs and LNs, as shown in Figure 6.

Each glomerulus does the following: Receives RN input from the antennae receptors upstream; inhibits other glomeruli within the AL via LNs; and sends excitatory signals downstream via Projection Neurons (PNs).

In general, each PN is innervated in a single glomerulus. In moths, there are ~5 PNs rooted in each glomerulus (60 glomeruli, ~300 PNs). The Network Model assumes all PNs from a given glomerulus carry the same signal (because they share the same glomerulus and therefore inputs, and perhaps also because of ephaptic binding) (Sjoholm, 2006).

Glomeruli also initiate pooled Inhibitory Projection Neurons (QNs) that send inhibitory signals downstream to the KCs.

The AL contains a powerful macro-glomerulal complex (MGC), which processes pheromone. Because pheromone response has fundamentally different dynamics than food odor response (Jefferis et al., 2007), the model ignores it. Only the glomeruli associated with non-pheromone (food) odors are modeled.

Connections in the AL are not plastic with long-term persistence (Dacks et al., 2012). While some evidence of short-term plasticity exists, the Network Model ignores this option.

#### 4.2.3. Lateral Horn

The LH receives input from the PNs. It then sends an inhibitory signal to the KCs. This inhibition from the LH appears to ensure that the KCs fire very sparsely and thus act as coincidence detectors for signals from the AL (Sjoholm, 2006; Gruntman and Turner, 2013; Lin et al., 2014).

The LH is also suspected of containing a parallel system for processing certain intrinsically-known odors in short-cut fashion (labeled lines) (Luo et al., 2010). Since this parallel system is (by definition) not involved with learning, the Network Model ignores it. The LH is modeled solely as a simple sparsifying inhibition on the KCs.

[Note: The locust and honeybee, which have more complex olfactory systems and different use-cases in terms of odor processing, have a time-oscillating depolarization mechanism (local potential fields, LPF) Perez-Orive et al., 2002 which serves a similar purpose to LH inhibition in the moth. LPF oscillations are absent in the moth Martin et al., 2011.]

#### 4.2.4. Mushroom Body and Kenyon Cells

The KCs (~4,000) in the MB are believed to encode odor memories in a high-dimensional, sparse space (Turner et al., 2008). Odors with no meaning to the moth still have non-zero codes in the KCs.

KCs receive excitatory input from the PNs and inhibitory input from QNs, both of which vary greatly between KCs, since each KC is innervated by only ~10 PNs (Martin et al., 2011). The connection map appears to be random (Caron et al., 2013). The KCs also receive generalized damping inhibition from the LH. (There is some evidence in drosophila of an MB → MB global inhibitory neuron Lin et al., 2014, with the same essential effect as LH inhibition; the Network Model excludes this possibility.) KCs fire very sparsely, generally respond to only a single odor, and are silent absent that odor (Honegger et al., 2011). KCs are treated as noise-free. Their output is an excitatory signal sent to the extrinsic neurons (ENs) Campbell et al. (2013).

In addition to olfactory input, the KCs receive input signals from other parts of the moth (e.g., hearing) (Sjoholm, 2006). Because the Network Model targets olfactory learning, it ignores these other inputs and uses a reduced number of KCs (~2,000 instead of ~4,000).

The synaptic connections in the MB (PNs → KCs, QNs → KCs, and KCs → ENs) are plastic, i.e., they can be modified during training (Menzel and Manz, 2005). The generalized inhibition from LH → KCs is modeled as non-plastic (actual physiology is not known). This LH inhibition is modeled as a global damping term on KCs, giving dynamics equation equivalent to the Pitt- McColloch approximation (McCulloch and Pitts, 1943) as used in Huerta and Nowotny (2009), Bazhenov et al. (2013), Mosqueiro and Huerta (2014), and peng2017.

#### 4.2.5. Extrinsic Neurons

Though located in the lobes of the MB, here ENs are not considered part of the MB, which is taken to be synonymous with the KCs. ENs are few in number compared to the KCs (~10s ) (Campbell et al., 2013; Hige et al., 2015). They are believed to be “readout” neurons, that interpret the KC codes as actionable signals (eg “approach,” “avoid”) (Masse et al., 2009). We assume that ENs trigger actions when their output FRs exceed some threshold.

We define Learning as: Permanently boosting EN responses beyond their naive (untrained) level, so that EN responses to reinforced stimuli can consistently exceed an action-triggering threshold. This is tantamount to modifying the moth's behavior.

#### 4.2.6. Octopamine (Reward Circuit)

A large neuron delivers octopamine to the entire AL and MB, in response to positive stimuli, e.g., sugar at the proboscis. It acts as a reward feedback to the system. A similar neuron delivers dopamine to the AL and MB in response to negative stimuli, and acts as an aversive feedback signal (Dacks et al., 2012). Learning does not occur without octopamine (or dopamine) (Hammer and Menzel, 1998).

Despite their opposite reward values, both octopamine and dopamine act in the same way when sprayed on a neuron: They increase the neuron's general tendency to fire (Riffell et al., 2012). In the Network Model this effect is modeled as making a neuron more responsive to excitatory inputs (e.g., from odors and RNs) and less responsive to inhibitory inputs (e.g., from LNs). Details of octopamine's effects if any on particular neural types are not well-characterized. In the Network Model octopamine directly affects RNs and LNs but not PNs in the AL (cf section 3.1); has no direct effect on KCs or ENs (though there are strong indirect effects); and has no effect on the LH inhibitory signal.

It is unclear whether octopamine delivery to both the MB and AL is necessary and sufficient for learning (Hammer and Menzel, 1998; Dacks et al., 2012). The Network Model assumes that octopamine controls an “on/off” switch for Hebbian growth, i.e., there is no plasticity in the MB (and therefore no learning) without octopamine.

### 4.3. Network Model Model Description

This section describes our Network Model model in detail. It covers the firing rate measure used to compare model output to *in vivo* data; model dynamics; plasticity and other details; model parameters; and moth generation. All coding was done in Matlab. Computer code for the Network Model in this paper will be found at: https://github.com/charlesDelahunt/SmartAsABug.

#### 4.3.1. Model Dynamics

Our Network Model uses standard firing rate dynamics (Dayan and Abbott, 2005) (chapter 7), evolved as stochastic differential equations (Higham., 2001). We use a firing rate model for two reasons. First, it is the simplest model able to both capture the key response and learning dynamics of the moth, and also allow calibration to our *in vivo* datasets. Our *in vivo* datasets are spike trains and thus admit use of an integrate-and-fire or Izhikevich model; but at the cost of more parameters, higher complexity, and longer simulation times, without (in our eyes) commensurate benefit. Second, and importantly, we wish to apply our results to engineered NNs, which makes a firing rate model the natural choice due to its close similarities to NNs.

The firing rate model is formulated as follows: Let *x*(*t*) = firing rate (FR) for a neuron. Then

(3) $$\begin{array}{r} \tau \frac{dx}{dt}=-x+s\left(\Sigma w_{i}u_{i}\right)=-x+s\left(w\cdot u\right),\text{where} \end{array}$$

**w** = connection weights;

**u** = upstream neuron FRs;

*s*() is a sigmoid function or similar.

PN dynamics are given here as an example. Full model dynamics are given in Supplementary Material. PNs are excitatory, and project forward from AL→MB:

(4) $$\begin{array}{r} \tau \frac{d\text{P}}{dt}=-\text{P}+s\left(\tilde{\text{P}}\right)+{dW}^{P}\text{where} \end{array}$$

*W*(*t*) = brownian motion process;

$\tilde{P}$=−(1−γ*o*(*t*)*M*^*OP*^)*M^LP^*u^*L*^+(1+*o*(*t*)*M*^*OP*^)*M^RP^*u^*R*^;

*M*^*OP*^ = octopamine → PN weight matrix (diagonal *nG*×*nG*);

*M*^*LP*^ = LN → PN weight matrix (*nG*×*nG* with *trM*^*LP*^ = 0);

*M*^*RP*^ = RN → PN weight matrix (diagonal *nG*×*nG*);

*o*(*t*) indicates if octopamine is active (*o*(*t*) = 1 during training, 0 otherwise).

**u**^*L*^ = LN FRs, vector *nG*×1;

**u**^*R*^ = RN FRs (*nG*×1);

*γ* = scaling factor for effects on inhibition.

#### 4.3.2. Discretization

The discretization uses Euler-Maruyama (E-M), a standard step-forward method for SDEs (Higham., 2001). Euler (i.e., noise-free): *x*_*n*+1_ = *x*_*n*_+Δ*tf*(*x*_*n*_) Euler-Maruyama: $x_{n+1}=x_{n}+\Delta tf\left(x_{n}\right)+\epsilon \text{randn(0,1)}\sqrt{\Delta t}$, where ϵ controls the noise intensity.

#### 4.3.3. Convergence

Timestep Δ*t* was chosen such that noise-free E-M evolution gives the same timecourses as Runge-Kutta (4th order), via Matlab's ode45 function. Δ*t* = 10 mSec suffices to match E-M evolution to R-K in noise-free moths. Values of Δ*t* ≤ 20 mS gives equivalent simulations in moths with AL noise calibrated to match *in vivo* data. Values of Δ*t*≥40 mS show differences in evolution outcomes given AL noise.

#### 4.3.4. Plasticity

The model assumes a Hebbian mechanism for growth in synaptic connection weights (Hebb, 1949; Cassenaer and Laurent, 2007). That is, the synaptic weight *w*_*ab*_ between two neurons *a* and *b* increases proportionally to the product of their firing rates (“fire together, wire together”): Δ*w*_*ab*_(*t*)∝*f*_*a*_(*t*)*f*_*b*_(*t*). Thus, synaptic plasticity is defined by:

(5) $$\begin{array}{r} \Delta w_{ab}\left(t\right)=\gamma f_{a}\left(t\right)f_{b}\left(t\right),\text{where}\gamma \text{is a growth parameter.} \end{array}$$

There are two layers of plastic synaptic weights, pre- and post-MB: AL→MB (*M*^*P, K*^, *M*^*Q, K*^), and MB→ENs (*M*^*K, E*^). Learning rate parameters of the Network Model were calibrated to match experimental effects of octopamine on PN firing rates and known moth learning speed (e.g., 4–8 trials to induce behavior modification) (Riffell et al., 2012). The Network Model does not decay unused synaptic weights. Training does not alter octopamine delivery strength matrices (*M*^*O*, *^). That is, the neuromodulator channels are not plastic (unlike, for example, the case in Grant et al., 2017).

#### 4.3.5. Odor and Octopamine Injections

Odors and octopamine are modeled as hamming windows. The smooth leading and trailing edges ensures low stiffness of the dynamic ODEs, and allows a 10 mS timestep to give accurate evolution of the SDEs in simulations.

#### 4.3.6. Training

Training on an odor consists of simultaneously applying stimulations of the odor, injecting octopamine, and “switching on” Hebbian growth. Training with 5–10 odor stimulations typically produces behavior change in live moths.

### 4.4. Firing Rate Measure

To compare PN firing rate statistics from *in vivo* experiments and from Network Model simulations (i.e., model calibration), we use a measure of firing rate (FR) based on Mahalanobis distance, similar to the measure $\frac{DF}{F}$ common in the literature (Silbering and Galizia, 2007; Turner et al., 2008; Campbell et al., 2013; Hong and Wilson, 2015). The premise is that neurons downstream respond to a ±1 std change in FRs equally (modulo different connection weights), independent of the sometimes large (up to 40x) magnitude differences in the raw spontaneous FRs of different neurons. The FR measure is defined as follows:

1. Each PN has a spontaneous firing rate (FR) with a gaussian noise envelope.
2. PNs with FR < 1 spike/sec are ignored, on the assumption that such PNs represent artifacts of experiment (also, the gaussian noise assumption fails). About 10% of PNs from *in vivo* data fall in this category.
3. Output FR activity of PNs is measured as *M*(*t*) = distance from mean spontaneous FR, in units of time-varying std dev of spontaneous FR (i.e., Mahalanobis distance): Let
  *F*(*t*) = raw firing rate (spikes per second).
  *S*(*t*) = spontaneous firing rate (no odor).
  *μS*(*t*) = moving average of S (no odor).
  $\bar{\mu }S\left(t\right)$ = smoothed estimate of the moving average *μS*, eg a quadratic or spline fit.
  *σ*_*S*_(*t*) = standard deviation of *S*, calculated using $S-\bar{\mu }S$ values within a moving window centered on *t*.
  *σ*_*S*_(*t*) and *μS*(*t*) are typically steady absent octopamine, but are often strongly modulated by octopamine.
  Then the measure of FR activity *M* is:
  (6) $$\begin{array}{r} M\left(t\right)=\frac{F\left(t\right)-\bar{\mu }S\left(t\right)}{\sigma_{S}\left(t\right)} \end{array}$$
4. *M* is related to the measure $\frac{DF}{F}$:
  $\frac{DF}{F}=\frac{\Delta F}{F}=\frac{F\left(t\right)-\mu S}{\mu S}$, i.e., $\frac{DF}{F}$ is change in FR, normalized by spontaneous FR. The key difference between *M* and $\frac{DF}{F}$ is whether or how *σ*_*S*_ is estimated, due to varying exigencies of experiment. Our experimental data allow reasonable estimates of *σ*_*S*_ and *μS*. Network Model simulations produce very good estimates, since computer models are more amenable to repeated trials than live moths.

### 4.5. Model Parameters

There is a risk, when modeling a system, of adding too many free parameters in an effort to fit the system. Fewer free parameters are better, for the sake of generality and to avoid overfitting. Conversely, we wish to reasonably match the physiological realities of the system. Because the key goal of this paper was to demonstrate that a simple model, in terms of parameters and structure, can reproduce the learning behavior of the AL-MB, we made efforts to minimize the number of free parameters. For example, neuron-to-neuron connections in the model are defined by their distributions, i.e., two parameters each. These are (usually) distinct for different source-to-target pairs (eg LN→RN, LN→LN, etc). Some mean and std dev parameters for distributions are shared among different neuron types (e.g., LNs, PNs, and QNs all share the same variance scaling parameter).

#### 4.5.1. Parameter List

The model has in total 47 free parameters:

1. Structure: 5 (e.g., number of neurons in each network)
2. Dynamics: 12 (noise: 2. decay and sigmoid: 3. Hebbian growth: 6. misc: 1).
3. Spontaneous RN FRs: 3.
4. Connection matrices: 27 (controlling non-zero connection ratios: 5; synaptic weights (eg *M*^*P, K*^, *M*^*R, P*^): means 12, std devs 4; octopamine weights (e.g., *M*^*O, R*^, *M*^*O, P*^): means 6, std devs 2).

#### 4.5.2. Dynamics Parameters

The differential equations of all neuron types share the same decay rate, set to allow return to equilibrium in ~1 s, consistent with *in vivo* data. Neurons also share parameters of the sigmoid function within the differential equation. Noise added via the SDE model is controlled by a single parameter ϵ, the same for all neuron types. It is determined by empirical constraint on $\frac{\sigma_{S}}{\mu S}$, as shown in column 2 of Figure 2.

#### 4.5.3. Connection Matrix Generation

Connection weight matrices (eg *M*^*P, K*^ etc) are generated in a standard way, from Gaussian distributions with std dev σ defined proportional to the mean *μ*, using a scaling factor *v*: $M^{*,*}\sim N\left(\mu_{c},\sigma_{c}^{2}\right)$ where *μ*_*c*_ depends on the neuron types being connected, and *σ*_*c*_ = *v*μ**_*c*_. Many connection types typically share the same *v*.

A special feature of the AL is that all the neurons in a given glomerulus share a common environment. For example, all the neurons, of whatever type, in glomerulus *A* will share the same strong (or weak) LN axon from glomerulus *B*. Thus, the RN, LN, and PNs in a given glomerulus are all correlated. In addition, neuron types are correlated. To model this dual set of correlations, connection matrices in the AL are generated as follows. As an example, consider LN connection matrices in the AL:

1. A glomerulus-glomerulus connection matrix *M*^*L, G*^ is created, which defines LN arborization at the glomerular level.
2. This connection matrix is multiplied by a neural type-specific value to give *M*^*L, P*^,*M*^*L, L*^, and *M*^*L, R*^ connection matrices. This is particularly important when tuning the various inhibitory effects of LNs on RNs, PNs (QNs), and LNs.
3. Sensitivity to GABA: A separate variance factor determines glomerular sensitivity to GABA (i.e., sensitivity to inhibition). This is tuned to match data in the literature Hong and Wilson (2015), and applies to LN-to-PN(QN) (i.e., *M*^*L, P*^) connections only.

The goal of this two-stage approach is to enforce two types of similarity found in the AL: (i) Connections to all neurons within a single glomerulus are correlated; and(ii) connections to all neurons of a certain type (LN, PN, RN) are correlated.

Due to constraints of the biological architecture there are many zero connections. For example, about 85% of entries in the AL→MB weight matrix are zero because MB neurons connect to only ~10 projection neurons (Caron et al., 2013). All MB→EN weights are set equal at the start of training. Training leads rapidly to non-uniform distributions as inactive connections decay and active connections strengthen.

#### 4.5.4. RN Spontaneous Firing Rates

RNs in the glomeruli of the AL have noisy spontaneous firing rates (Bhandawat et al., 2007). The Network Model simulates this by assigning spontaneous firing rates to RNs. These spontaneous firing rates are drawn from a gamma distribution plus a bias: γ(*x*|α, β, *b*) = *b*+$\frac{\beta^{\alpha }}{\Gamma \left(\alpha \right)}x^{\alpha -1}e^{-\beta x}$, where α, β are shape and rate parameters, and Γ(·) is the Gamma function. This can be thought of as a source of energy injected into the system, at the furthest upstream point (absent odor). Other energy sources are odor signals and octopamine. The spontaneous firing rates of all other neurons in the Network Model are the result of their dynamics as RN spontaneous FRs propagate through the system.

### 4.6. Discrepancies Between Biology and Model

There are some known discrepancies between our Network Model and the moth AL-MB. These are listed below.

#### 4.6.1. Connection Weight Distributions

This model version uses gaussian distributions to generate initial connection weights. However, moths used in live experiments are older and thus presumably have modified PN → KC and KC → EN connection weights. If this modification was strong, we might expect the connection weight distributions to tend toward a scale-free rather than gaussian distribution (Barabasi and Albert, 1999). This represents an unknown discrepancy between structure parameters of the live moths used in experiments vs the model.

#### 4.6.2. Hebbian Pruning

The Network Model contains no pruning mechanism to offset, via decay, the Hebbian growth mechanism. Such pruning mechanisms are common in nature, so it is reasonable to suppose that one might exist in the AL-MB. The moth has inhibitory as well as excitatory feed-forward connections from AL to MB. In the Network Model, pruning is functionally replaced by Hebbian growth of QN → KC inhibitory connections, which act to inhibit KCs and thus offset the growth of excitatory PN → KC connections (this does not directly offset KC → EN Hebbian growth). Thus omitting a separate Hebbian decay mechanism is a matter of convenience rather than a match to known biology.

#### 4.6.3. Non-olfactory Input to KCs

In addition to olfactory input, the KCs receive signals from other parts of the moth, eg hearing. Because this model targets only olfactory learning, it ignores these other inputs to the KCs, and reduces the total number of KCs (from ~4,000 to ~2,000).

#### 4.6.4. Number of QNs

There are believed to be about 3–6 QNs projecting from the AL to the MB. This model sets their number at about 15. The reason is that, absent a Hebbian pruning system in the model, the QNs function as the brake on runaway increases in KC responses due to Hebbian growth. So the increased number of QNs is a compensation for the lack of a weight-decay system.

#### 4.6.5. Number of ENs

This model version has only one EN, since its goal is to demonstrate simple learning. The moth itself possesses multiple ENs.

#### 4.6.6. LH Inhibition

The LH → KC inhibitory mechanism used in this chapter is modeled as a time-invariant global signal, delivered equally to all KCs. This simplifies the model parameter space while retaining the essential functionality of the LH. A more refined version of LH → KC inhibition might vary in strength according to PN output, since the same PN signals that excite the KCs also excite the LH. The actual dynamics of the AL → LH → KC linkage are not known, beyond the principle that inhibition from the LH sparsifies the KC codes and makes the individual KCs act as coincidence detectors.

### 4.7. *In vivo* Neural Recordings Data

Model parameters were calibrated by matching Network Model performance to *in vivo* electrode readings from the ALs of live moths. The various performance metrics are described in section 2.

**Figure 7:**
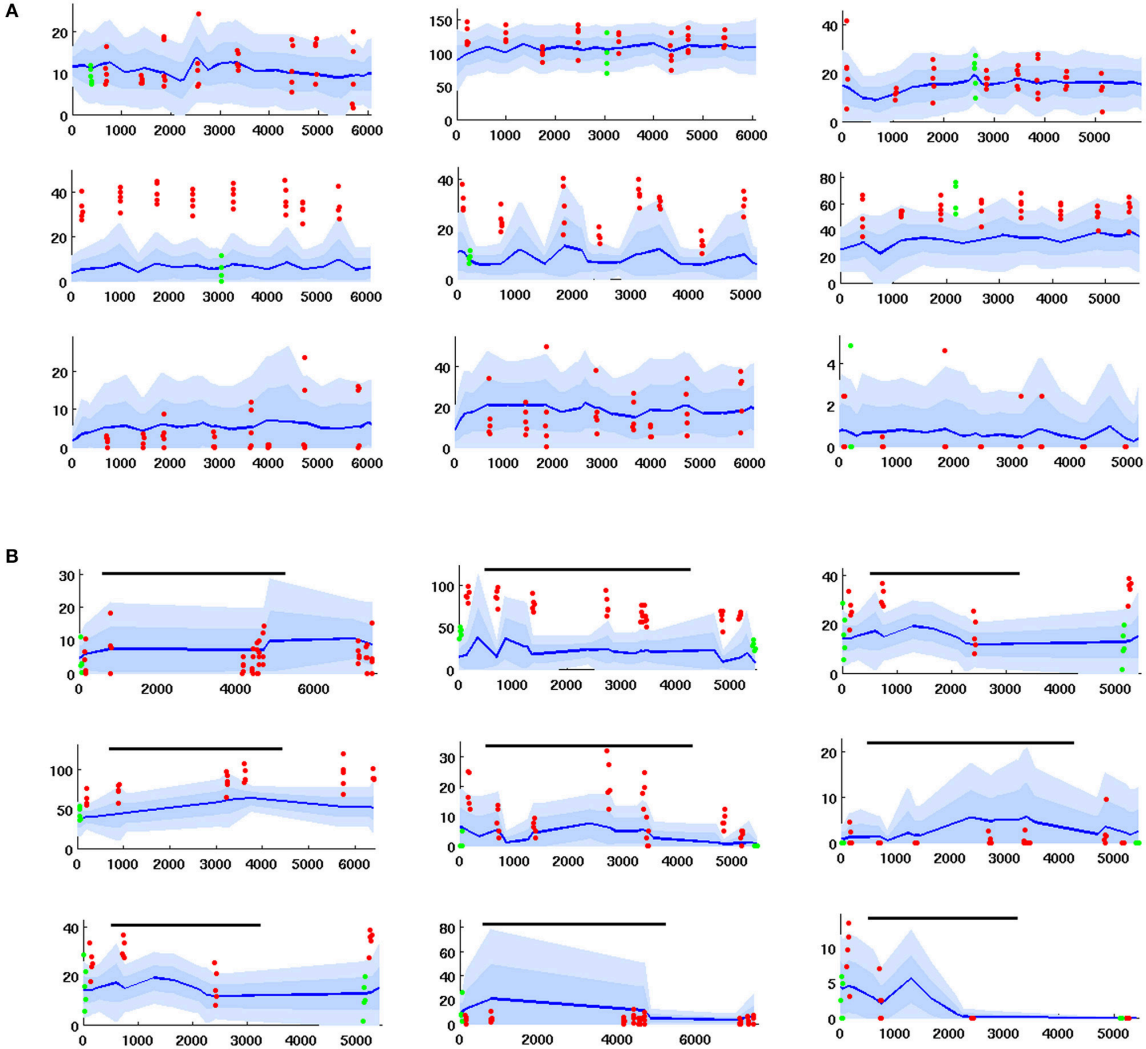
Time series of PN firing rates from *in vivo* experiments. x-axis = time, y-axis = FR. Blue lines = mean spontaneous rate, shaded regions = ±1 and 2 std. Red dots are odor responses. Green dots are response to control (mineral oil). **(A)** PN response, given odor plus coincident sugar reward, ie plus octopamine (time series for PNs with odor only are similar, but with less strong odor responses). Top row: unresponsive to odor. Middle row: excited response to odor. Bottom row: inhibited response to odor. **(B)** PNs with octopamine wash added in mid-experiment, then rinsed away (duration shown by black line). Octopamine can alter (up, down, or not at all) the spontaneous FR and/or the odor response, so there are 9 possible modulation regimes. This grid of timecourses shows a typical PN from each regime. Top row: spontaneous FR in unaffected. Middle row: spontaneous FR is boosted. Bottom row: spontaneous FR is inhibited. First column: odor response is unaffected. Second column: odor response is boosted. Third column: odor response is inhibited.

Electrode data was collected by the lab of Prof Jeff Riffell (Dept of Biology, UW). It consists of timecourses of PN firing rates measured via electrode in the AL of live moths, during a variety of regimes including:

1. Series of 0.2 s odor stimulations delivered without octopamine. These experiments gave data re PN response to odor relative to PN spontaneous (baseline) FRs, absent octopamine.
2. Series of 0.2 s odor stimulations delivered coincident with sugar reward (which delivers octopamine). This gave data re how PN odor response is modulated by octopamine, relative to octopamine-free spontaneous FR. see Figure 7A.
3. Series of 0.2 s odor stimulations, delivered first without and then coincident with an octopamine wash applied to the AL. This gave data re how PN spontaneous FR and PN odor response are modulated by octopamine. see Figure 7B.

In most cases the applied odor consisted of a collection of 5 volatiles, which taken together stimulate many glomeruli in the AL. It was selected to ensure sufficient odor-responsive PNs, such that inserted electrodes would detect interesting (i.e., responsive) PNs. Further details re *in vivo* data collection can be found in Shlizerman et al. (2014) and in Supplementary Material. Example timecourses are shown in Figure 7.

### 4.8. Simulation Setup

For Network Model learning experiments, the time sequence of events for simulations, shown in Figure 1, is as follows:

1. A period of no stimulus, to assess baseline spontaneous behavior.
2. Four odor stimuli are delivered, 16 stimulations each (two odors were used in MB sparseness experiments).
3. A period of control octopamine, i.e., without odor or Hebbian training.
4. The system is trained (odor + octopamine + Hebbian mechanism) on one of the odors.
5. A period of no stimulus, to assess post-training spontaneous behavior.
6. The odors are re-applied (16 stimulations each), without octopamine, to assess effects of training on odor response.

## 5. Glossary

**Antennal lobe (AL)**: A collection of neurons innervated by odor receptors in the antennae. It sends signals to the mushroom body via projection neurons. Connections in the AL are not plastic.

**Mushroom body (MB)**: A collection of neurons (Kenyon cells–KCs) downstream from the antenna lobe. The MB is believed to store odor codes that serve as a memory, allowing the moth to recognize odors. Connections in the MB are plastic.

**Lateral horn (LH)**: A collection of neurons which receives input from the AL and sends inhibitory output to the MB. One of its roles is to enforce sparse firing in MB neurons.

**Receptor neuron (RN)**: These neurons respond to odors (volatiles) at the antennae and stimulate the antenna lobe. RNs respond to different, distinct odors.

**Glomerulus**: The antenna lobe is divided into about 60 glomeruli, each of which is a self-contained collection of neurons (projection and lateral), innervated by RNs that respond to particular odors.

**Projection neuron (PN)**: Each glomerulus contains projection neurons, whose output innervates the KCs and also the lateral horn, but not other glomeruli in the AL, i.e., they are feed-forward only. Most PNs start in one glomerulus and are excitatory. A few PNs arborize in several glomeruli and are inhibitory (we refer to inhibitory PNs as “QNs”). Each glomerulus initiates about five PNs.

**Lateral neuron (LN)**: Each glomerulus contains lateral neurons, which innervate other glomeruli in the AL. LNs are inhibitory. One function is competitive inhibition among glomeruli. Another function is gain control, i.e., boosting low signals and damping high signals.

**Kenyon cell (KC)**: Neurons in the calyx of the MB. These have very low FRs, and tend to respond to particular combinations of PNs. KCs respond sparsely to a given odor. There are about 4,000 KCs, i.e., a two-orders-of-magnitude increase over the number of glomeruli. Each KC synapses with about ten PNs. Connections into and out of KCs are plastic.

**Extrinsic neuron (EN)**: A small number of neurons downstream from the KCs. ENs are thought to be “readout” neurons. They interpret the odor codes of the KCs, deciding to eg “ignore,” “approach,” or “avoid”.

**Firing rate (FR)**: The number of spikes/second at which a neuron fires. Typically FRs are counted using a window (e.g., 500 ms). The moth's response to odor stimulations is episodic, with FR spikes in FR and rapid return to spontaneous FRs. Neurons respond to relative changes in FR, rather than to raw magnitude changes. A neuron's relative change in FR is scaled by its spontaneous FR (see section 4.4 below).

**Octopamine**: A neuromodulator which stimulates neural firing. The moth spritzes octopamine on both the AL and MB in response to sugar, as a feedback reward mechanism. Dopamine has a similar stimulating effect on both AL and MB, but it reinforces adverse rather than positive events.

## Data Availability Statement

The *in vivo* datasets analyzed in this study will be found at: https://github.com/charlesDelahunt/SmartAsABug.

## Author Contributions

CD developed the model, conducted experiments, and wrote the draft. JR provided data and domain expertise, and reviewed the draft. JK guided the research, and contributed to and reviewed the draft.

### Conflict of interest statement

The authors declare that the research was conducted in the absence of any commercial or financial relationships that could be construed as a potential conflict of interest.
