# Supplementary material for: Biological Mechanisms for Learning: A Computational Model of Olfactory Learning in the Manduca sexta Moth, With Applications to Neural Nets
Source: Front Comput Neurosci. 2018 Dec 19;12:102. doi: 10.3389/fncom.2018.00102 (PMC6306094; doi:10.3389/fncom.2018.00102)
Supplement: Supplementary file 1 [file Data_Sheet_1.pdf]

Supplementary Material: Biological Mechanisms for Learning: A Computational Model of Olfactory Learning in the *Manduca sexta* Moth, with Applications to Neural Nets

1 MOTH ANTENNA LOBE NEURAL RECORDING DATASETS

1.1 Multichannel Recording Methods

Adult male *M. sexta* were reared in the laboratory at the University of Washington on an artificial diet under a long-day (17/7 hr light/dark cycle) photoperiod and prepared for experiments 2–3 d after emergence. In preparation for recording, the moth was secured in a plastic tube with dental wax, and the caudal end of the head capsule was opened and the piece of cuticle that is the attachment site of the pharyngeal dilator muscles is positioned forward and readhered to the headcapsule with myristic acid. The proboscis was extended and adhered to a piece of teflon tubing, 7-cm in length, that allowed movement and extension of the proboscis along its length and at the tip. Behavioral response to odor stimuli was determined by the percentage of proboscis movement response between treatments, allowing for a conservative estimate of a learned response. This preparation allows full access to the exposed AL while having no effect on the moth's ability to feed normally. During recordings, the brain was superfused slowly with physiological saline solution (in mM: 150 NaCl, 3 CaCl2, 3 KCl, 10 N-tris[hydroxymethyl] methyl-2 aminoethanesulfonic acid buffer, and 25 sucrose, pH 6.9) throughout the experiment.

Olfactory stimuli were delivered to the preparation by pulses of air from a constant air stream were diverted through a glass syringe containing a piece of filter paper bearing odor stimuli. The stimulus was pulsed by means of a solenoid-activated valve controlled by the SciWorks acquisition software (Data Wave Technologies, Longmont, CO, USA). The outlet of the stimulus syringe was positioned 2 cm from and orthogonal to the center of the antennal flagellum ipsilateral to the ALs.

We used a classic conditioning paradigm to examine the effects on AL neurons while the moth learned to associate an odor with a sugar reward. Odor stimuli were delivered in a three second pulses, and one second after odor onset, the unconditioned stimulus (US; 1 uL of 20% sucrose solution) was applied to the proboscis for ca. 2 s. A ten minute inter-trial interval separated each training trial, and moths were trained over eight trials. After the conditioning trials were completed, a test trial was performed during which only the trained odor was presented to assess the behavioral and odor-driven ensemble responses as a result of the conditioning treatment.

AL Recordings were made with 16-channel silicon multielectrode recording arrays (a4x4-3mm50-177; NeuroNexus Technologies, Ann Arbor, MI, USA). The spatial distribution design of the recording array suits the dimensions of the AL in *M. sexta*, with four shanks spaced 125 um apart, and each with four recording sites 50 um apart. The four shanks were oriented in a line parallel to the antennal nerve. The probe was advanced slowly through the AL using a micromanipulator (Leica Microsystems, Bannockburn, IL, USA) until the uppermost recording sites were just below the surface of the AL. Extracellular activity was

acquired with a RZ2 base station (Tucker-Davis Technologies, Alachua, FL, USA) and a RP2.1 real time processor (Tucker-Davis Technologies) and extracellular activity in the form of action potentials, or spikes, were extracted from the recorded signals and digitized at 25 kHz using the Tucker-Davis Technologies data-acquisition software.

Threshold and gain settings were adjusted independently for each channel, and spikes were captured in the 4-channel, or 'tetrode', recording configuration: any spike that passed threshold on one channel triggered the capture of spikes recorded on the other 3 channels on the same shank. Offline Sorter v.3 (Plexon Neurotechnology Research Systems, Dallas, TX, USA) was used to sort extracellular spikes based on their waveform shape and spikes were assigned timestamps to create raster plots and calculate peri-stimulus time histograms (PSTH). Only those clusters that were separated in three dimensional space (PC1–PC3) after statistical verification (multivariate ANOVA; $P < 0.05$) were used for further analysis (typically 6–18 units were isolated per ensemble). Each spike in each cluster was time-stamped, and these data were used to create raster plots and to calculate peristimulus time histograms (PSTHs), interspike interval histograms, cross-correlograms, and rate histograms.

1.2 List of *in vivo* data sets

1. AL, odor only: PNs, one odor, no octopamine. 7 preps with 8 - 16 PNs each.
2. AL, odor + octopamine. PNs, one odor, sugar reward. 10 preps with 9 - 21 PNs each.
3. AL + MB, odor + octopamine. PNs and KCs, one odor, sugar reward. 1 prep, with 7 PNs and 12 KCs.
4. AL, odor + octo wash: PNs, one odor, octopamine directly applied to AL. 7 preps: 6 preps with 8 - 13 PNs each; 1 prep with one pheromone-responsive neuron
5. AL, odors only (BEA): PNs, several odors and concentrations. 12 preps with 14 - 17 PNs each.
6. AL, odors only (ESO): PNs, several odors and concentrations. 4 preps with 12 - 14 PNs each.

2 FULL EQUATIONS OF MODEL DYNAMICS

2.1 Differential equations by neuron type

$$\tau_{\mathrm{R}} \cdot d\mathbf{u}^{\mathrm{R}} = f_{\mathrm{R}}\big(\mathbf{u}^{\mathrm{R}}, \mathbf{u}^{\mathrm{L}}, \mathbf{u}^{\mathrm{S}}, M^{\mathrm{L,R}}, M^{\mathrm{S,R}}, M^{\mathrm{O,R}}, o(t)\big) \ + \ d\mathbf{W}^{\mathrm{R}} \tag{S1}$$

$$\tau_{\mathrm{P}} \cdot d\mathbf{u}^{\mathrm{P}} = f_{\mathrm{P}}\big(\mathbf{u}^{\mathrm{R}}, \mathbf{u}^{\mathrm{P}}, \mathbf{u}^{\mathrm{L}}, M^{\mathrm{L,P}}, M^{\mathrm{R,P}}, M^{\mathrm{O,P}}, o(t)\big) \ + \ d\mathbf{W}^{\mathrm{P}} \tag{S2}$$

$$\tau_{\mathrm{Q}} \cdot d\mathbf{u}^{\mathrm{Q}} = f_{\mathrm{Q}}\big(\mathbf{u}^{\mathrm{R}}, \mathbf{u}^{\mathrm{Q}}, \mathbf{u}^{\mathrm{L}}, M^{\mathrm{L,Q}}, M^{\mathrm{R,Q}}, M^{\mathrm{O,Q}}, o(t)\big) \ + \ d\mathbf{W}^{\mathrm{Q}} \tag{S3}$$

$$\tau_{\mathrm{L}} \cdot d\mathbf{u}^{\mathrm{L}} = f_{\mathrm{L}}\big(\mathbf{u}^{\mathrm{R}}, \mathbf{u}^{\mathrm{L}}, M^{\mathrm{L,L}}, M^{\mathrm{R,L}}, M^{\mathrm{O,L}}, o(t)\big) \ + \ d\mathbf{W}^{\mathrm{L}} \tag{S4}$$

$$\tau_{\mathrm{K}} \cdot d\mathbf{u}^{\mathrm{K}} = f_{\mathrm{K}}\big(\mathbf{u}^{\mathrm{P}}, \mathbf{u}^{\mathrm{Q}}, \mathbf{u}^{\mathrm{D}}, M^{\mathrm{P,K}}, M^{\mathrm{Q,K}}\big) \ + \ d\mathbf{W}^{\mathrm{K}} \tag{S5}$$

$$\tau_{\mathrm{E}} \cdot d\mathbf{u}^{\mathrm{E}} = f_{\mathrm{E}}\big(\mathbf{u}^{\mathrm{K}}, \mathbf{u}^{\mathrm{E}}, M^{\mathrm{K,E}}\big) \tag{S6}$$

where

$$\begin{cases} f_{\mathrm{R}} = -\mathbf{u}^{\mathrm{R}} + \mathrm{sigmoid}\big[-\big(I - \gamma \cdot o(t) \cdot M^{\mathrm{O,R}}\big) M^{\mathrm{L,R}}\, \mathbf{u}^{\mathrm{L}} \ + \ \big(I + o(t) \cdot M^{\mathrm{O,R}}\big) M^{\mathrm{S,R}}\, \mathbf{u}^{\mathrm{S}}\big] \\ f_{\mathrm{P}} = -\mathbf{u}^{\mathrm{P}} + \mathrm{sigmoid}\big[-\big(I - \gamma \cdot o(t) \cdot M^{\mathrm{O,P}}\big) M^{\mathrm{L,P}}\, \mathbf{u}^{\mathrm{L}} \ + \ \big(I + o(t) \cdot M^{\mathrm{O,P}}\big) M^{\mathrm{R,P}}\, \mathbf{u}^{\mathrm{R}}\big] \\ f_{\mathrm{Q}} = -\mathbf{u}^{\mathrm{Q}} + \mathrm{sigmoid}\big[-\big(I - \gamma \cdot o(t) \cdot M^{\mathrm{O,Q}}\big) M^{\mathrm{L,Q}}\, \mathbf{u}^{\mathrm{L}} \ + \ \big(I + o(t) \cdot M^{\mathrm{O,Q}}\big) M^{\mathrm{R,Q}}\, \mathbf{u}^{\mathrm{R}}\big] \\ f_{\mathrm{L}} = -\mathbf{u}^{\mathrm{L}} + \mathrm{sigmoid}\big[-\big(I - \gamma \cdot o(t) \cdot M^{\mathrm{O,L}}\big) M^{\mathrm{L,L}}\, \mathbf{u}^{\mathrm{L}} \ + \ \big(I + o(t) \cdot M^{\mathrm{O,L}}\big) M^{\mathrm{R,L}}\, \mathbf{u}^{\mathrm{R}}\big] \\ f_{\mathrm{K}} = -\mathbf{u}^{\mathrm{K}} + \mathrm{sigmoid}\big[-\big(\mathbf{u}^{\mathrm{D}} + M^{\mathrm{Q,K}}\, \mathbf{u}^{\mathrm{Q}}\big) \ + \ M^{\mathrm{P,K}}\, \mathbf{u}^{\mathrm{P}}\big] \\ f_{\mathrm{E}} = -\mathbf{u}^{\mathrm{E}} + M^{\mathrm{K,E}}\, \mathbf{u}^{\mathrm{K}} \end{cases}$$

2.2 Key to variables and parameters

Symbol	Type	Size/Value	Description and Remarks
R	superscript		Refers to the *receptor neurons* subpopulation.
P	superscript		Refers to the *excitatory projection neurons* subpopulation.
Q	superscript		Refers to the *inhibitory projection neurons* subpopulation.
L	superscript		Refers to the *lateral neurons* subpopulation.
K	superscript		Refers to the *kenyon cells* subpopulation.
E	superscript		Refers to the readout *extrinsic neurons* subpopulation.
O	superscript		Refers to the *octopamine* neurotransmitter.
nG	scalar	60	Number of glomeruli in the antenna lobe. *
nS	scalar	4	Number of different stimuli (odors).
nQ	scalar		Number of inhibitory projection neurons.
nK	scalar	2000	Number of kenyon cells.
nE	scalar	1	Number of extrinsic neurons.

Symbol	Type	Size/Value	Description and Remarks
$\mathbf{u}^{\mathrm{R}}$	vector	$nG \times 1$	FRs of the receptor neurons subpopulation.
$\mathbf{u}^{\mathrm{P}}$	vector	$nG \times 1$	FRs of the exc. projection neurons subpopulation.
$\mathbf{u}^{\mathrm{Q}}$	vector	$nQ \times 1$	FRs of the inh. projection neurons subpopulation.
$\mathbf{u}^{\mathrm{L}}$	vector	$nG \times 1$	FRs of the lateral neurons subpopulation.
$\mathbf{u}^{\mathrm{K}}$	vector	$nK \times 1$	FRs of the kenyon cells subpopulation. Sparse.
$\mathbf{u}^{\mathrm{E}}$	vector	$nE \times 1$	FRs of the extrinsic neurons subpopulation.
$\mathbf{u}^{\mathrm{S}}$	vector	4×1	Magnitudes of stimulus odors.
$\mathbf{u}^{\mathrm{D}}$	vector	$nK \times 1$	Damping from the Lateral Horn.
$M^{\mathrm{S,R}}$	matrix	$nG \times nS$	Stimulus $\rightarrow \mathbf{u}^{\mathrm{R}}$ connections.
$M^{\mathrm{O,R}}$	matrix	$nG \times nG$	Octopamine $\rightarrow \mathbf{u}^{\mathrm{R}}$ connections. Diagonal matrix.
$M^{\mathrm{O,L}}$	matrix	$nG \times nG$	Octopamine $\rightarrow \mathbf{u}^{\mathrm{L}}$ connections. Diagonal matrix.
$M^{\mathrm{R,L}}$	matrix	$nG \times nG$	Connection weights $\mathbf{u}^{\mathrm{R}} \rightarrow \mathbf{u}^{\mathrm{L}}$.
$M^{\mathrm{R,P}}$	matrix	$nG \times nG$	Connection weights $\mathbf{u}^{\mathrm{R}} \rightarrow \mathbf{u}^{\mathrm{P}}$. Diagonal matrix.
$M^{\mathrm{R,Q}}$	matrix	$nQ \times nG$	Connection weights $\mathbf{u}^{\mathrm{R}} \rightarrow \mathbf{u}^{\mathrm{Q}}$.
$M^{\mathrm{P,K}}$	matrix	$nK \times nG$	Connection weights $\mathbf{u}^{\mathrm{P}} \rightarrow \mathbf{u}^{\mathrm{K}}$.
$M^{\mathrm{Q,K}}$	matrix	$nK \times nQ$	Connection weights $\mathbf{u}^{\mathrm{Q}} \rightarrow \mathbf{u}^{\mathrm{K}}$.
$M^{\mathrm{L,R}}$	matrix	$nG \times nG$	Connection weights $\mathbf{u}^{\mathrm{L}} \rightarrow \mathbf{u}^{\mathrm{R}}$.
$M^{\mathrm{L,P}}$	matrix	$nG \times nG$	Connection weights $\mathbf{u}^{\mathrm{L}} \rightarrow \mathbf{u}^{\mathrm{P}}$.
$M^{\mathrm{L,Q}}$	matrix	$nQ \times nG$	Connection weights $\mathbf{u}^{\mathrm{L}} \rightarrow \mathbf{u}^{\mathrm{Q}}$.
$M^{\mathrm{L,L}}$	matrix	$nG \times nG$	Connection weights $\mathbf{u}^{\mathrm{L}} \rightarrow \mathbf{u}^{\mathrm{L}}$.
$M^{\mathrm{K,E}}$	matrix	$nE \times nK$	Connection weights $\mathbf{u}^{\mathrm{K}} \rightarrow \mathbf{u}^{\mathrm{E}}$.
$o(t)$	function	0 or 1	Flags when octopamine is active (typically during training).
γ	scalar	0.5	Scaling factor for octopamine's effects on inhibition. *
τ_{R}	scalar		
τ_{P}	scalar		
τ_{Q}	scalar		
τ_{L}	scalar		
τ_{K}	scalar		
τ_{E}	scalar		

* Each glomerulus receives one RN and one octopamine input, and initiates one PN and one LN.
* Octopamine decreases the response to inhibition less than it increases the response to excitation

3 ANOVA ANALYSIS OF NETWORK MODEL LEARNING

The differential increase in EN response to trained vs control odors was almost always significant to $p < 0.01$. When odors' naive EN response magnitudes differed by ratio > 3, sometimes differences in either raw increases or percentage increases (not both) did not attain this level of significance, while the other metric did. An example of learning results when odors' naive EN response magnitudes differ widely is shown in Figure S1.

The p-values of 336 trained odor/control odor pairs are plotted in Figure S2, against the ratio of their mean naive odor responses $\frac{\mu_T}{\mu_C}$, for 28 moths randomly generated from a template, with three control odors and one trained odor. Each p-value is for the trained odor vs one control odor (so there are 12 data points per moth). Column 1 shows p-values for the change in raw EN response, as in Figure S1 (C), trained vs control. Trained odors with very low-magnitude naive response often did not have raw increases larger than high-magnitude control odors. Column 2 shows p-values for the percentage change in EN response, as in Figure S1 (B), trained vs control.

Unless the naive EN responses for the two odors were highly disparate (eg by factor of $>$3x), the differential increase in EN response of the trained vs control odors is almost always significant, measured both as raw and as percentage. Figure S3 plots the percentage of 336 trained-control pairs that had p-values for both measures of EN response increase (ie as raw and as percentage) below the listed threshold (eg $p = 0.01$), for 336 trained-control pairs whose ratio ($\frac{\mu_T}{\mu_C}$ or $\frac{\mu_C}{\mu_T}$) is within the bound given on the x-axis. Figure S3 shows how many moths, generated from template with no constraint on unbalanced naive odor EN responses, had differential post-training EN responses with significance $p < 0.01$, for both measures (as raw and as a percentage).

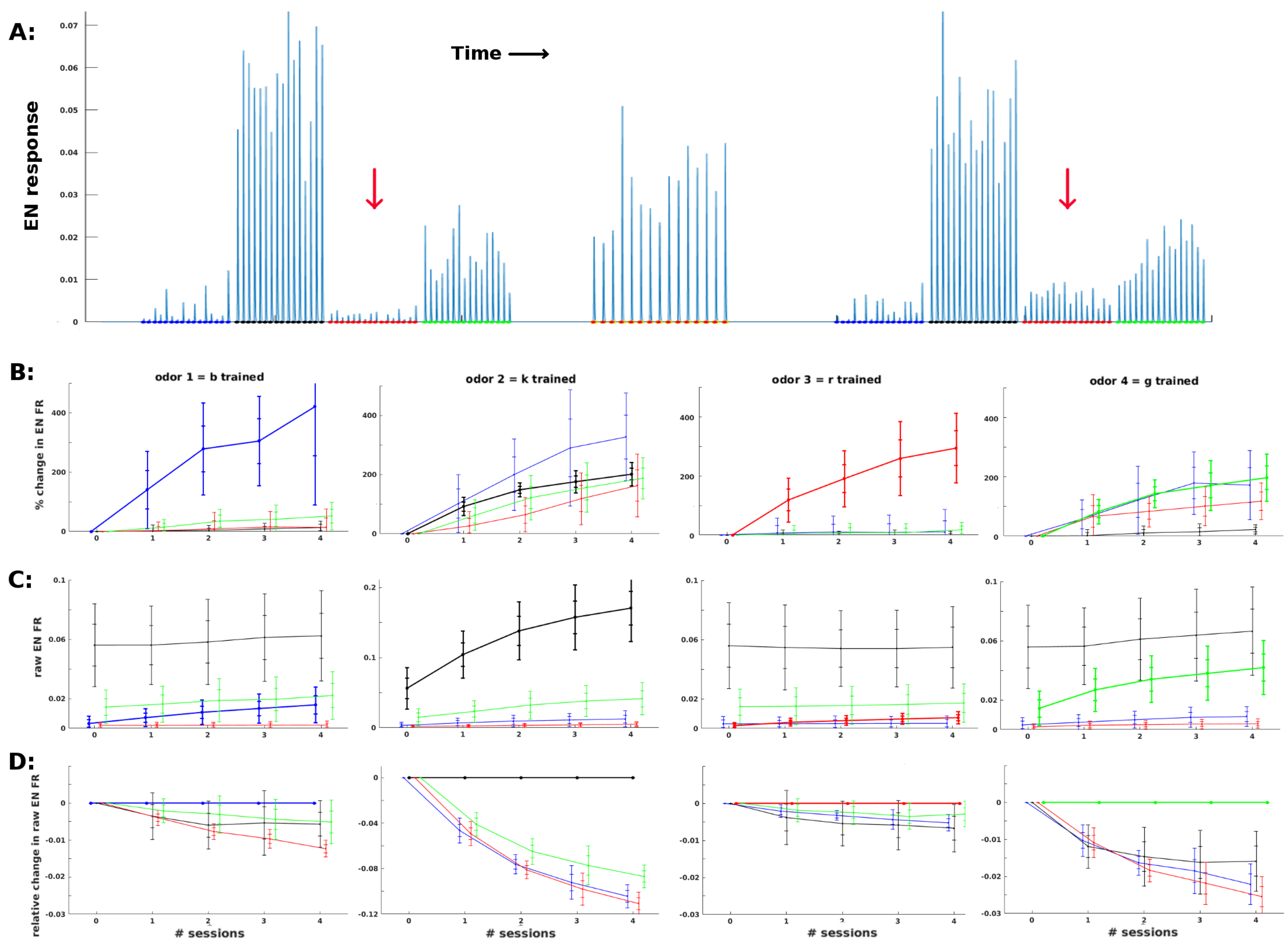

Figure S1. **Effect of training on EN FRs, given odors with unequal naive response magnitudes.** When odors induced naive EN responses of very different magnitudes, then trained odor response increased much more than control odor responses either in raw magnitude, or as a percentage, or both.
A: Typical timecourse showing magnitudes EN responses before and after training the third (red) odor, indicated by red arrow, over 15 odor stimulations. This corresponds to the third column in panels B - D, at index 3 on the x-axis. Note that only the third (red) odor's EN response changes magnitude.
Panels B - D: Changes to ENs during training. x-axis = number of training sessions. Each column shows results of training a given odor, color coded: blue, black, red. y-axis measures raw EN or percent change in EN. 21 trials per data point.
B: Percent change (from pre-training) in ENs, mean ± 2 stds.
C: Raw EN FRs, mean ± 2 stds.
D: Changes in raw EN FRs, normalized by trained odor (ie subtract the trained odor's changes from all odors), mean ± 2 std devs. This shows how far each control odor lagged behind the trained odor.
Note that the trained odor dominates in either raw increase (panels C, D) if naive response to trained odor was large, or in percent increase (panel B) if naive response to trained odor was small.

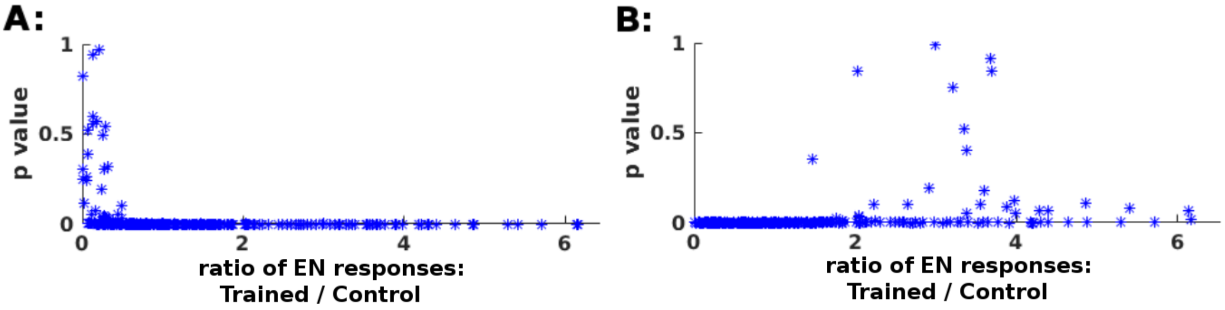

Figure S2. ***p*-values for trained-control odor pairs:** For raw and percentage change in EN responses. *p*-values are sometimes high (for one metric or the other) when trained and control odors have highly disparately-scaled naive responses μ_T (= mean raw T) μ_C (= mean raw C). **A:** *p*-values for change in raw EN responses. **B:** *p*-values for percentage change in EN responses.
When μ_T is larger (right end of x-axis), the *p*-value for raw change (A) is consistently very low, but the *p*-value for percentage change (B) can be high, since even a small incidental change to a low-intensity odor can be a large percentage change. When μ_C is larger (left end of x-axis), the *p*-value for percentage change (B) is consistently very low, but the *p*-value for raw change (A) can be high, since even a small percentage change to a high-response odor corresponds to a large raw change. When naive odor responses are roughly matched, eg within 3x (ie 0.33 to 3), *p*-values for both raw and percentage change are very low. 20 training stimulations per datapoint.

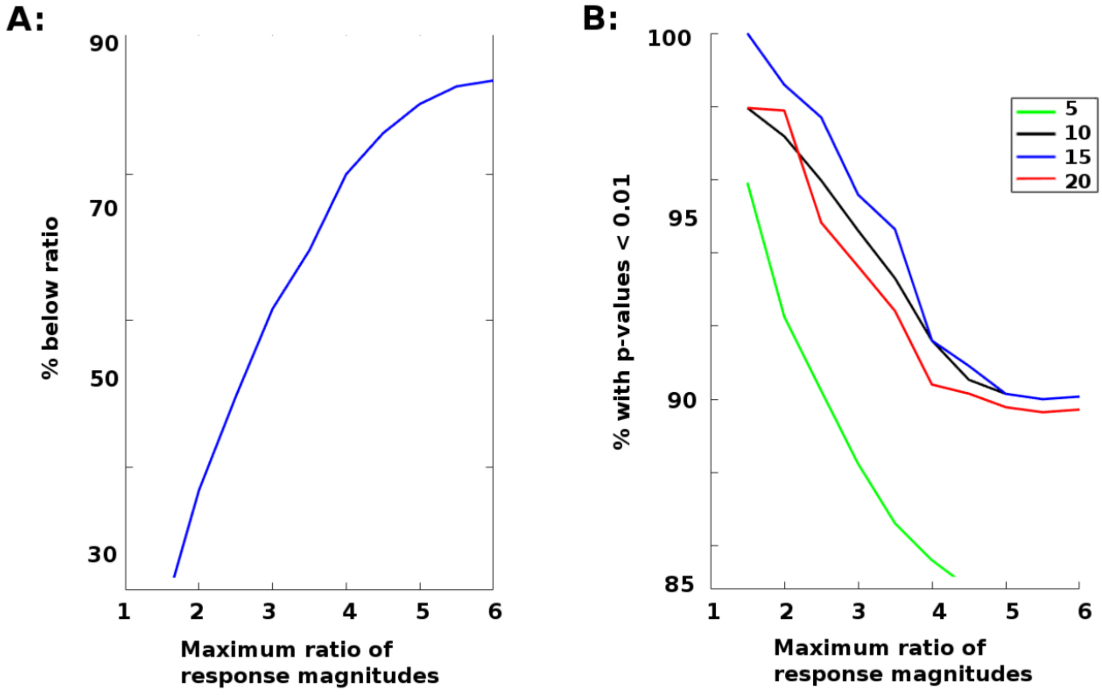

Figure S3. **Fractions of *p*-values below 0.01 for trained-control odor pairs** In most cases, the trained odor shows much larger increases in EN response magnitude (thus most *p*-values are low).
A: The percentage of trained-control odor pairs with EN response magnitudes within the ratios given on the x-axis.
B: The percentage of trained-control pairs, with EN response magnitudes within the ratios given on the x-axis, whose training-induced changes in EN responses were distinct with *p*-value < 0.01. Each curve is for a different number of training stimulations. More training increases distinctions, up to 15 stimulations. But additional training actually hinders distinctions, as control odor response reinforcement begins to overtake trained odor reinforcement.
